# Dissecting the chromatin interactome of microRNA genes

**DOI:** 10.1093/nar/gkt1294

**Published:** 2013-12-18

**Authors:** Dijun Chen, Liang-Yu Fu, Zhao Zhang, Guoliang Li, Hang Zhang, Li Jiang, Andrew P. Harrison, Hugh P. Shanahan, Christian Klukas, Hong-Yu Zhang, Yijun Ruan, Ling-Ling Chen, Ming Chen

**Affiliations:** ^1^Department of Bioinformatics, College of Life Sciences, Zhejiang University, Hangzhou 310058, P. R. China, ^2^Center for Bioinformatics, College of Life Science and Technology, Huazhong Agricultural University, Wuhan 430070, P.R. China, ^3^Department of Molecular Genetics, Leibniz Institute of Plant Genetics and Crop Plant Research Gatersleben (IPK), Corrensstrasse 3, D-06466 Gatersleben, Germany, ^4^The Jackson Laboratory for Genomic Medicine, and Department of Genetic and Development Biology, University of Connecticut, 400 Farmington, Connecticut 06030, USA, ^5^Department of Mathematical Sciences and School of Biological Sciences, University of Essex, Colchester, Essex CO4 3SQ, UK and ^6^Department of Computer Science, Royal Holloway, University of London, Egham, Surrey, TW20 0EX, UK

## Abstract

Our knowledge of the role of higher-order chromatin structures in transcription of microRNA genes (*MIRs*) is evolving rapidly. Here we investigate the effect of 3D architecture of chromatin on the transcriptional regulation of *MIRs*. We demonstrate that *MIRs* have transcriptional features that are similar to protein-coding genes. RNA polymerase II–associated ChIA-PET data reveal that many groups of *MIRs* and protein-coding genes are organized into functionally compartmentalized chromatin communities and undergo coordinated expression when their genomic loci are spatially colocated. We observe that *MIRs* display widespread communication in those transcriptionally active communities. Moreover, miRNA–target interactions are significantly enriched among communities with functional homogeneity while depleted from the same community from which they originated, suggesting *MIRs* coordinating function-related pathways at posttranscriptional level. Further investigation demonstrates the existence of spatial *MIR–MIR* chromatin interacting networks. We show that groups of spatially coordinated *MIRs* are frequently from the same family and involved in the same disease category. The spatial interaction network possesses both common and cell-specific subnetwork modules that result from the spatial organization of chromatin within different cell types. Together, our study unveils an entirely unexplored layer of *MIR* regulation throughout the human genome that links the spatial coordination of *MIRs* to their co-expression and function.

## INTRODUCTION

MicroRNAs (miRNAs) are a large family of small noncoding RNAs (∼21 nt) that have emerged as key posttranscriptional regulators of gene expression in eukaryotic organisms. More than 1500 miRNA genes (*MIRs*) have been identified in the human genome (1) and they likely regulate the activity of more than half of all protein-coding genes (2). Functional investigations indicate that these *MIRs* control various developmental and cellular processes, and the dysregulation of their expression is being found to be associated with diverse human diseases, including cancers (3,4).

Despite these great advances in our recognition of the important biological roles of *MIRs*, our understanding of the transcriptional regulation of *MIRs* is still developing. It is generally believed that the transcription of most *MIRs* is mediated by RNA polymerase II (RNAPII) (5). There have been several exceptional cases of *MIRs* reported to be transcribed by RNAPIII (6-9). However, some of these putative RNAPIII-transcribed *MIRs* (for example, *mir-565*, *mir-886* and *mir-1975*) are actually other types of RNAs, such as tRNAs, Y RNAs and Vault RNAs, which are transcribed by RNAPIII (10–13), while others (for example, chromosome 19 *MIR* cluster, C19MC) displayed no occupancy by RNAPIII (7,8) but showed evidence of being transcribed by RNAPII instead (14). These misannotated *MIRs* have been subsequently removed from miRBase (1) ahead of further investigation.

*MIRs* originate from precursor molecules (pri-miRNAs). These transcripts can be encoded as independent transcription units (TUs), in polycistronic clusters or within the introns of protein-coding genes (15,16), and contain poly(A) tails as well as cap structures (17). Approximately 50% of human *MIRs* are organized into introns of protein-coding genes (intragenic *MIRs*) and are likely transcribed in parallel with their host transcripts (18), whereas *MIRs* located within intergenic regions (intergenic *MIRs*) are believed to be derived from independent TUs (15).

Transcriptional regulation is not only determined by the DNA code at the linear chromosomal level. It also involves additional layers of higher-order chromosomal organization, which provides the chromatin context that can either facilitate or block the initiation of transcription (19). In particular, the development of chromosome conformation capture (3C) and similar techniques (20) have demonstrated that the spatial organization of chromatin has important transcriptional roles in regulating gene expression (21). Recent observations of RNAPII-associated chromatin interactions using ChIA-PET (22) showed that the transcription of protein-coding genes could be coordinated spatially through extensive promoter–promoter chromatin interactions in close proximity. Because the transcription of *MIRs* is RNAPII-mediated, the availability of a genome-wide RNAPII-associated chromatin interaction provides us the opportunities to study, on a genome scale, how 3D chromatin interaction affects the transcription regulation of *MIRs*.

In this study, we have integrated comprehensive 3D chromatin interaction data (22) and genome-wide histone modification and expression data sets (Supplementary Table S1) from the Encyclopedia of DNA Elements (ENCODE) project (23). We establish a potential mechanistic link between chromatin-associated spatial interactions and transcriptional regulation of *MIRs* by RNAPII (Supplementary Figure S1).

## MATERIALS AND METHODS

### Overview of the integrated data analysis strategy

The huge genome-wide data sets from the ENCODE project (23) provide us with an unprecedented opportunity to dissect the underlying mechanisms of chromatin organization and its impact on transcriptional regulation and gene expression using an integrative approach. In this study, we have performed integrative data analysis to investigate the relationships between the spatial organization of the human miRNAome, the local chromatin status and how it affects *MIR* regulation (Supplementary Figure S1). We began our analysis with the identification of putative *MIR* promoters using integrative data sources and prediction methods (see below). We then used ChIA-PET with RNAPII peak data and ChIP-seq of histone modifications and DNA methylation data to characterize the chromatin features of *MIRs*. We also correlated the chromatin status with gene expression data, and examined the expression patterns between *MIRs* and their nearby protein-coding genes. Next, we developed a statistical model to assign *MIRs* into different chromatin interaction models based on ChIA-PET interaction data, and thus obtained a global *MIR*–*MIR* interaction network. Finally, we integrated RNA-seq data and disease information to systemically uncover the relationship between chromatin organization, cell-specific *MIR* regulation and disease biology.

In this study, we focus our analysis on K562 (chronic myelogenous leukemia) and MCF7 (mammary gland, adenocarcinoma) cell lines (see http://encodeproject.org/ENCODE/cellTypes.html for detailed information). The data sources used in this analysis are available in Supplementary Table S1 and summarized as below.

### Data sources

*Human protein-coding genes and functional information*. The human (*Homo sapiens*) protein-coding genes with HGNC (symbol from the HUGO Gene Nomenclature Committee) symbols were downloaded from Ensembl (http://www.ensembl.org/; release 65) and RefSeq database (http://www.ncbi.nlm.nih.gov/RefSeq/). The gene ontology (GO) annotation and ID mapping data were retrieved from Ensembl using the BioMart tool. The best-curated list of known disorder–gene associations was obtained from Online Mendelian Inheritance in Man database (http://omim.org/; December 2012). We only considered entries with the ‘(3)’ tag, for which there is strong evidence that at least one mutation in the particular gene is causative to the disorder. Subsequently, we manually classified each disorder into 20 primary disorder classes, according to the classification scheme described in (24).

*Human MIRs and their disease annotation*. The human *MIR* annotation information was retrieved from the miRBase database (http://www.mirbase.org/; release 18) (1). *MIRs* are grouped into either ‘intragenic’ or ‘intergenic’ according to whether their genomic position overlaps existing gene models. Specially, pre-miRNAs embedded into annotated genes with the same strand are referred as ‘intragenic *MIRs*’, whereas pre-miRNAs located between genes are ‘intergenic *MIRs*’. The information about disease-related *MIRs* was obtained from the miR2Disease (25) and PhenomiR (26) databases.

*ChIA-PET data*. The RNAPII-associated ChIA-PET data were retrieved from (22), the ENCODE data repository site (http://genome.ucsc.edu/ENCODE/) and NCBI/GEO (GSE33664). The ChIA-PET data can be used to simultaneously identify protein binding sites and chromatin interactions in a whole-genome, *de novo* and unbiased manner (27). The ChIA-PET peaks reflect the binding intensity by RNAPII, while the interactions determine the genome-wide long-range chromatin contact map linked by RNAPII. We used both types of the data here to study the transcriptional regulation of both protein-coding and *MIRs*.

*RNA-seq data*. The RNA-seq data sets for protein-coding genes and small RNA-seq data sets for *MIRs* were downloaded from the ENCODE data repository site (http://genome.ucsc.edu/ENCODE/). The mapped files (in bam format) were directly downloaded from the ENCODE Web site and were subjected to expression estimation. The expression levels in FPKM (fragments per kilobase of exon per million fragments mapped) estimated by Cufflinks (28) were averaged for each mRNA when there were multiple replicates available. For *MIRs*, their abundances were measured in terms of RPM (reads per million total small RNA reads) using small RNA-seq data.

*Epigenetic modification data*. DNA methylation data as well as ChIP-seq data for histone modifications were also retrieved from the ENCODE data repository site (http://genome.ucsc.edu/ENCODE/). To characterize the chromatin features, such as histone modifications, transcription factor bindings, DNase I hypersensitive sites and DNA methylation, for the transcriptional regulation of *MIRs*, we used the epigenetic mark data to measure their profile around *MIR* promoters. For each location (the predicted promoters; see below), the number of peaks or tags within ±5 kb from the center of the locations were counted.

*Hi-C data*. The processed topological domains for each chromosome were obtained from a recent Hi-C study (29), which provided higher-coverage experiments. Domain boundaries were identified using a hidden Markov model at the 40-kb resolution (combined data set, http://chromosome.sdsc.edu/mouse/hi-c).

*miRNA-target interaction data*. Predicted targets of miRNAs were retrieved from six databases: TargetScan (30), miRanda (31), miRDB (32), PicTar (33), DIANA-microT (34) and MicroCosm (1). To consider highly confident miRNA–target interactions, only interactions supported by at least two databases were used in this analysis.

### Annotation of *MIR* promoters

**Figure 1.**
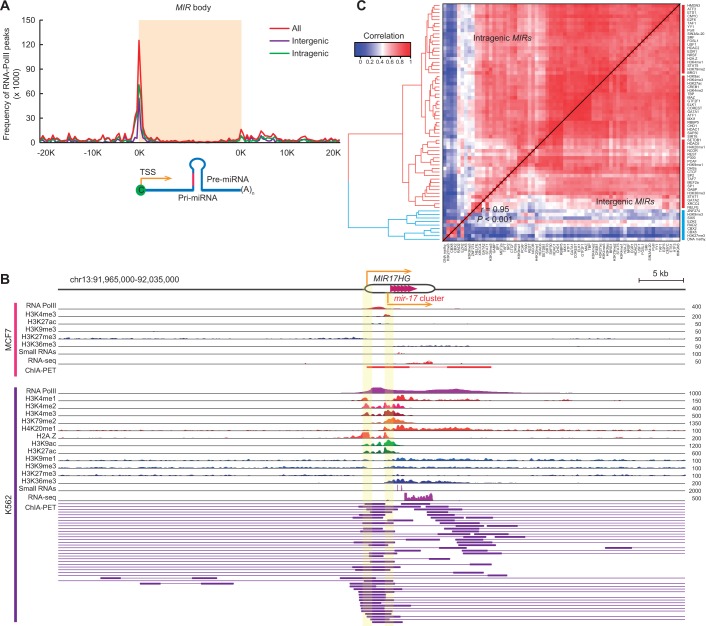
Epigenetic regulation of *MIRs*. (**A**) RNAPII binding peaks around *MIR* body. Intergenic (purple) and intragenic (green) *MIRs* are shown, respectively. (**B**) Chromatin features, RNAPII-associated ChIA-PET data and expression of the *mir-17* cluster. Predicted promoters of this *MIR* cluster and its host gene are highlighted. Data from MCF7 (top panel) and K562 cells (bottom panel) are shown, respectively. Only a partial list of chromatin markers is shown and a complete list can be found on the UCSC Genome Browser (http://genome.ucsc.edu/). (**C**) Heatmap matrix of pairwise Spearman correlations of modification profile of distinct chromatin features for *MIRs* in K562 cells. The upper diagonal shows the correlation coefficients based on intragenic *MIRs*. The lower diagonal shows the correlation coefficients based on intergenic *MIRs*. Heatmap plot was organized by hierarchical clustering with the tree (left) using Spearman’s correlation distances based on all *MIRs*. There is no observed difference of the chromatin modification patterns between intergenic and intragenic *MIRs* [Pearson correlation *r* = 0.95 and *P* < 0.001, Mantel test (41)]. See also Supplementary Figures S2, S4 and S5.

We annotated the *MIR* promoters or transcriptional start sites (TSSs) by integrating three different data sources (Supplementary Figure S2A). Firstly, the predicted TSSs were retrieved from the miRStart database (http://mirstart.mbc.nctu.edu.tw/) (35), which systematically incorporates high-throughput sequencing data derived from TSS-relevant experiments to identify TSSs of *MIRs*. We obtained 832 high-confidence *MIR* TSSs from this web resource. For the rest of the *MIRs*, we searched the putative TSSs within the 50-kb-long upstream region of each pre-miRNA by using DeepCAGE tags (36) from the FANTOM web resource (http://fantom.gsc.riken.jp/; FANTOM4) (37). This led to the identification of another 274 *MIR* TSSs. We also predicted the promoter regions and TSSs of *MIRs* using genome-wide RNAPII binding peaks derived from ChIA-PET data (22), which is based on the previous observation that RNAPII binding peaks are proximal to the TSS of *MIRs* (38). The nearest RNAPII peak within 50 kb upstream of pre-miRNA was assigned to the TSS of that *MIR*. We identified 180 additional *MIR* TSSs based on RNAPII-associated ChIA-PET peak data (Supplementary Figure S2A). Previous studies have shown that H3K4me3 can be considered as a useful chromatin mark for identifying active *MIR* promoters (9,39), we thus manually checked the putative promoter regions of these newly identified *MIRs* using H3K4me3 data (Figure 1B). Intragenic *MIR* may have their own promoters or share promoters with their host genes. To confirm whether the closest RNAPII peak is the TSS of a *MIR*, we included promoter-associated histone markers, such as H3K4me2, H3K4me3, H3K9ac and H3K27ac (40), as additional evidences, on the basis that promoter-associated markers and RNAPII peak were in the same positions around the site and closest to the associated *MIR*. In total, we identified 1286 *MIRs* with predicted promoters. If the TSS of a *MIR* lies within ±2-kb region around the TSSs of nearby protein-coding genes, this *MIR* was considered to share promoters with nearby genes. Otherwise, the *MIR* was considered to have an isolated promoter. Of all the annotated *MIRs*, 536 (35.2%) showed shared promoters with nearby protein-coding genes. Several examples can be found in Supplementary Figure S3. The full list of *MIR* TSSs is used for further analysis and provided in Supplementary Data Set S1. The RNAPII binding peak intensity within ±2 kb from the *MIR* TSS site is available as Supplementary Data Set S2.

### Construction of RNAPII-associated chromatin interaction network

Because *MIRs* and protein-coding genes are both transcribed by RNAPII, we used these two kinds of genes to construct a transcription-associated chromatin interaction network. The RNAPII-associated ChIA-PET interaction data in K562 and MCF7 cell lines were used to define the chromatin interaction network (Supplementary Figure S6A). Some detected ChIA-PET interactions may result from cancer-related genome translocation. We, therefore, removed genomic regions that mapped proximal to genomic structural variations in their respective genomes (42) before constructing the network. Next, for each ChIA-PET interaction (termed as ‘duplex interaction’), two sets of genes (including both protein-coding genes and *MIRs*) within ±2 kb from their predicted TSS sites to the two interacting anchor boundaries are considered to be chromatin linked to each other. These linked genes form complex interaction networks based on the connectivity of overlapping anchors from one duplex interaction to the others. We did this analysis for each replicate of the samples separately and for each type of cell lines (K562 cell line with three replicates and MCF7 with four replicates). To avoid gene interactions detected by chance, each pair of interacting genes should be recovered by at least two replicates. The whole chromatin interaction network consists of 2292 modules, which were referred to as chromatin communities (Supplementary Figure S6B). The full list of chromatin communities is provided in Supplementary Data Set S3.

### Analysis of *MIR*-related chromatin interaction models

To define *MIR*-related chromatin interaction models, we focus on only *MIRs* in the whole network. Each interacting *MIR* pair should be supported by at least two independent replicates, unless they are neighbors on the genome (in which case they should be recovered by at least one replicate), as neighboring interactions are believed to be more reliable to detect in ChIA-PET technology (27) and adjacent *MIRs* (in clusters) prefer to be transcribed together (15). It is notable that, in this case, two *MIRs* are linked together either ‘directly’ by shared chromatin interactions or ‘indirectly’ via some intermediate interactions. We detected 202 pairs of directly *MIR*–*MIR* interactions, most (98.0%) of which were intrachromosomal and 83.3% of which were supported by at least two replicates. Although most (62.4%) interchromosomal interactions were linked indirectly, they were relatively reliable, as they were supported by at least two independent data sets. We also incorporated RNAPII-associated ChIA-PET peak data to investigate transcriptional regulation of *MIRs* that are not assigned to the defined interaction models. We assigned all the annotated *MIRs* to three interaction models based on how the *MIRs* were involved in the interaction networks: (i) ‘basal transcription’ model in which *MIR* overlapped with standalone RNAPII peaks in its promoter regions, but did not overlap with any interaction anchors; (ii) ‘*MIR*-related chromatin interaction’ model that involved a *MIR*(s) that interacted with other *MIRs* or protein-coding genes; while (iii) other ‘not assigned’ model in which the promoters of the *MIRs* were not supported by any RNAPII peaks or interactions (Supplementary Figure S8A). We found 293 (19%) *MIRs* involved in the basal transcription model and nearly half of the *MIRs* (734; 48%) in the ‘*MIR*-related chromatin interaction’ models (Supplementary Figure S8B). The list of *MIR*-related chromatin interaction models can be found in Supplementary Data Set S4.

### Association analysis of chromatin communities with miRNA–target interactions

We outlined the strategy in Figure 3A. In brief, highly reliable miRNA–target pairs (see the above ‘Data sources’ section) were firstly mapped to chromatin interaction networks. There were 87 303 (21.4%) pairs in total mapped on the network (Supplementary Data Set S5). However, only 0.2% (160) of the mapped pairs was to arise from within the same communities. We observed that 104 (65%) of these self-interacting pairs were from the giant community, indicating targets show significant underrepresentation within the same community that ‘their’ *MIRs* come from, compared with that of by chance (*P* = 3.4 × 10^−^^9^, Fisher's exact test). To associate significantly overrepresented chromatin community interactions with the mapping of miRNA–target pairs, two communities were considered to be linked if there were at least 10 mapped miRNA–target interactions between them. The statistical significance of their interactions was tested by a hypergeometric test corrected for multiple comparison by a false discovery rate (FDR) at 0.001 level. The criterion of at least 10 mapped miRNA-target pairs between two communities is based on the observation that each target in the chromatin interaction network was predicted to be targeted by 10 *MIRs* on averages. With an extension analysis, we relax this criterion (for example, ≥5 interactions, Supplementary Figure S9A), and performed the analysis analogous to that shown in Figure 3. We observed that (i) the overall layout of the community network remain largely unaltered; (ii) miRNA–target interactions are enriched between function-related communities; and (iii) miRNA–target interactions are preferentially depleted from within common communities. Overall, the main findings of the current study are robust to the relaxation of the criteria we used in our analysis.

### Random control analyses

We performed several random control analyses during this study. The basis was to extract the same number of genes/interactions chosen randomly from the same background sample, and then to calculate similar properties. To obtain significant statistics, we performed 10^3^ independent randomizations for each analysis. The Mann–Whitney U test was used to test the significance of distributions between the random control and observed data sets, and to calculate the *P*-value. Specifically, (i) to obtain the random control of the distributions of miRNA–target pairs between and within communities in Figure 3B, we chose the same number of protein-coding genes randomly for each community (nothing to do with *MIRs*). We then calculated the percentage of genes targeted by *MIRs* between or within communities. (ii) To assess the significance of function similarity between communities linked by miRNA–target pairs in Figure 3E, we performed GO enrichment analysis for each community and calculated the number of shared over-presented terms for random control and observed samples. (iii) To test the significance of *MIR*–*MIR* interactions from the same family or disease category in Figure 5B and C, we randomly generated the same number interactions from the *MIR* interactome and then calculated the distribution. FDR values were calculated from the 10^3^ random data sets as the probability of random values significantly greater than observed values (with the Mann–Whitney U test *P* < 0.001).

### Statistical analysis and data visualization

Most statistical analyses in this study were performed by using the R statistical package (http://www.r-project.org/; release 2.14.1). GO term enrichment analysis was performed using hypergeometric test corrected for multiple comparison by the Benjamini–Hochberg method (FDR < 0.05), implemented in GO::TermFinder software (43).

Most of the networks presented in this study were visualized using Cytoscape platform (44) with force-directed layouts. The network properties, such as scale-freeness, were performed using Network-Analyzer plug-in in Cytoscape.

For visualization purposes, the Integrative Genomics Viewer (IGV) (45) was used to view the interaction PET data and various peak data; Circos (46) was used to view in the *MIR* interaction map across the whole genome.

## RESULTS

### Transcriptional properties of *MIRs*

We first examined the transcription properties of *MIRs* using RNAPII-associated ChIA-PET data (22). The RNAPII peak data provided a rich source for predicting *MIR* promoters (see ‘Materials and Methods’ section). Based on searching RNAPII peaks within 50 kb upstream of pre-miRNAs, we identified 180 novel putative *MIR* promoters (Supplementary Figure S2A), which were not supported by other public databases (35,37,47). In total, we obtained 1286 (84.4%) *MIRs* with predicted promoters (Supplementary Data Set S1). From the combined data sets from K562 and MCF7 cell lines, we found that nearly two-thirds (66%; 343 intergenic and 658 intragenic *MIRs*) of the annotated *MIRs* showed high-confidence RNAPII binding sites around their predicted promoter regions (Supplementary Figure S2B and C and Supplementary Data Set S2). Most of these *MIRs* associated binding peaks are enriched around the predicted transcription start sites (TSSs) of the *MIRs* (Figure 1A). Similar observations were separately seen for intergenic and intragenic *MIRs*. Specially, we observed a high correlation between the distribution of RNAPII binding peaks in intragenic *MIRs* and the gene expression of their host protein-coding genes (Pearson correlation *r* = 0.98; *P* < 2.2 × 10^−^^16^; Supplementary Figure S2D), indicating that most, if not all, intragenic *MIRs* are co-transcribed with their host genes (see Supplementary Figure S3A and B for specific examples). For some intergenic *MIRs*, they have their own promoters for transcription (Supplementary Figure S3C and D). Some intergenic *MIRs* share common promoters with their nearby protein-coding genes, suggesting co-transcription (Supplementary Figure S3E–G). Notably, *MIRs* and nearby genes with opposite directions of transcription showed higher correlation of RNAPII binding distributions (*r* = 0.62) as compared with those with identical directions (*r* = 0.39; Supplementary Figure S2E), suggesting the importance of the bidirectional arrangement of promoters as a regulatory mechanism within the human genome (48).

Next, we determined whether *MIRs* associated with higher RNAPII binding peaks were more likely to be actively transcribed. We analyzed the transcriptional levels measured by small RNA-seq reads from ENCODE (23) and observed that the binding intensity at promoter sites correlated well with the expression level of the corresponding *MIRs* (Supplementary Figure S2F). The higher the RNAPII occupancy, the higher the *MIR* expression level tends to be (Supplementary Figure S2G and H). Besides, we observed that *MIRs* and their host (for intragenic *MIRs*) and nearby protein-coding genes (for intergenic *MIRs*) had coordinated expression output (Supplementary Figure S2I).

In addition to transcriptional regulation directed by RNAPII, *MIRs* could also be subject to epigenetic control at the chromatin level, similar to that seen for protein-coding genes. We observed high enrichment of active chromatin marks, such as histone modifications including H3K4me3, H3K4me2, H3K4me1 as well as histone acetylation and histone variant H2A.Z, within *MIR* promoter regions, that correlated well with RNAPII binding and expression output (Figure 1B and Supplementary Figure S4). By contrast, when these marks were weakened or totally lost, the binding peaks and expression levels changed accordingly.

To further explore the association of chromatin state dynamics with RNAPII-associated binding intensity, we systemically examined a comprehensive list of currently available chromatin marks and functional binding sites from the ENCODE consortium (see ‘Materials and Methods’ section). An unbiased pairwise association analysis was carried out on data sets from the K562 cell line. Heatmaps organized by hierarchical clustering revealed two clear groups of correlated pairs that distinguished active chromatin marks (red) from repressive marks (blue), where active marks were positively correlated with RNAPII peaks (Figure 1C). Interestingly, the distribution of binding peaks for RNAPII closely followed the binding peaks of histone marks (such as H3K4me2, H3K4me3, H3K9ac, H3K27ac, H3K79me2, H3K4me1 and H2A.Z; as active promoters and/or strong enhancers), the elements bound with chromatin remodeling factors (such as BRG1 and CHD1), the binding sites for the chromatin insulator CTCF and DNase hypersensitive sites. In contrast, repressive histone modifications (such as H3K27me3 and H3K9me3), DNA methylation and bindings of polycomb proteins (such as CBX2 and CBX8), known to be present in broad domains that encompass inactive genes, showed opposite distribution patterns compared with that of RNAPII binding. To rule out the possibility that the expression of intragenic *MIRs* is just tracking the expression of their host protein-coding genes being regulated by the epigenetic factors, we performed association analysis for intergenic and intragenic *MIRs* separately. We observed that the overall patterns are consistent [*r* = 0.95 and *P* < 0.001, Mantel test (41); Figure 1C]. Collectively, our observations are in line with the emerging view derived from the surveys of protein-coding genes (*r* = 0.98 and *P* < 0.001, Mantel test; Supplementary Figure S5A), namely that the enrichment of active chromatin modifications positively correlates with RNAPII occupancy in promoter regions (49). This supports the hypothesis that, like protein-coding genes, *MIRs* are extensively regulated at the chromatin level.

### Transcription-associated chromatin interaction networks involved in both coding genes and *MIRs*

Our recent study (22) focusing on protein-coding genes revealed that discrete gene loci are frequently transcribed together within a transcription factory—a self-assembling and organizing nuclear structure, where RNAPIIs are concentrated and engaged in transcription (50). Because *MIRs* possess similar transcriptional properties to those of protein-coding genes, it leads us to ask whether they are transcribed together from the same factory. To our knowledge, the role of *MIRs* in such transcription-associated chromatin modules has not been probed in previous studies. To address this problem, we used RNAPII-associated ChIA-PET data from K562 and MCF7 cells (22) to construct a transcription-associated chromatin interaction network whose nodes represent the annotated genes (including both *MIRs* and protein-coding genes) and edges denote high confident chromatin interactions among these genes (Supplementary Figure S6A). We required that each gene pair should be recovered in at least two independent samples (see ‘Materials and Methods’ section). The whole network (Supplementary Figure S6B and Supplementary Data Set S3) consists of 18 414 nodes with 62 495 links sharing among the nodes, and the network structure is largely conserved between K562 and MCF7 cell lines (Supplementary Figure S6C). The network is organized as 2292 discrete network modules (referred to as chromatin communities hereafter), 80% of which (with at least five genes) are common to both cell types (Supplementary Figure S6D). Network topology analysis revealed that distributions of the community size (Supplementary Figure S6E) and node degree (Supplementary Figure S6F) exhibit a hallmark of scale-freeness. These findings are in line with a recent study (51) that used similar data sets but did not include *MIRs* within its analysis.

To investigate the spatial organization of these chromatin communities, we took the recent Hi-C data (29) into account. We found that genes (including both *MIRs* and protein-coding genes) from the same chromatin community identified by ChIA-PET data are frequently present in the topological associated domains (TADs) identified by Hi-C experiments (Supplementary Figure S7). The overall correlation is highly stable across different cell types. The observation of multiple TADs associating with the same chromatin community suggests that these domains were organized into higher spatial structures by RNAPII for co-transcription. Besides, we noticed that *MIRs* are frequently observed in the community-associated TADs (Supplementary Figure S7).

To elucidate the links between expression patterns of *MIRs* and their chromatin regulation, we assigned all the annotated *MIRs* into three chromatin models (22) based on the RNAPII-associated ChIA-PET data: (i) ‘basal transcription’ model, (ii) ‘*MIR*-related chromatin interaction’ model and (iii) ‘not assigned’ (see ‘Materials and Methods’ section; Supplementary Figure S8A and Supplementary Data Set S4). These chromatin models showed both common and cell-specific manners in the two cell types (Supplementary Figure S8B). We found that *MIRs* associated with chromatin interactions, especially those in multi-*MIR* interaction models, showed significantly higher expression levels than those in basal transcription models. However, as a control, *MIRs* without supporting RNAPII-associated ChIA-PET data had the lowest expression levels (Supplementary Figure S8C). These findings were the same for both K562 and MCF7 cell types. Altogether, our study of *MIRs*, along with other observations of protein-coding genes (22), suggest that spatially coordinated transcription factories exist in the nucleus and cooperatively transcribe both protein-coding genes and *MIRs*.

### Widespread involvement of *MIRs* in functionally compartmentalized chromatin communities

**Figure 2.**
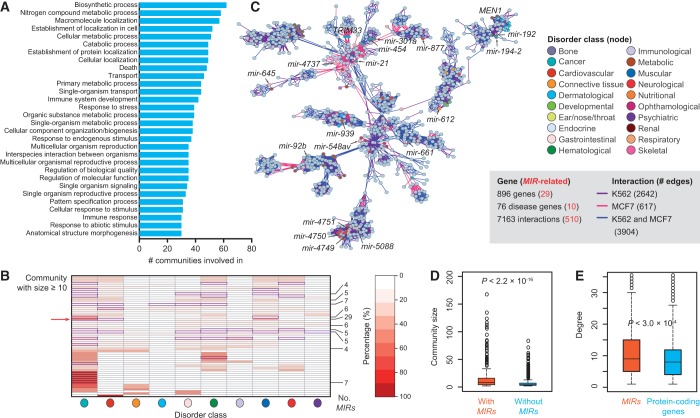
Widespread involvement of miRNA genes in functionally compartmentalized chromatin communities. (**A**) Top 30 GO biological process terms enriched in chromatin communities. Histogram shows the number of communities that a specific GO term is involved in. (**B**) Heatmap showing enrichment of disease classes for miRNA genes (*MIRs*) in communities with size ≥10. Rows present communities and column denote disease classes. The number of *MIRs* (No. *MIRs* >3) in each community is labeled on right. The significant enrichment categories (*P* < 0.001, χ^2^ test on categories with at least two *MIRs*) are indicated with solid boxes in violet. The arrow (left) indicates the largest chromatin community as shown in C. The ten disease classes from left to right are “Cancer,” “Cardiovascular”, “Connectivetissue,” “Dermatological,” “Gastrointestinal”, “Hematological”, “Immunological”, “Muscular”, “Neurological” and “Psychiatric”. (**C**) Network designating the largest chromatin community. Nodes (representing protein-coding genes or *MIRs*) are colored according to their disease classification (24) and shown in pie chart (gray indicate non-disease genes). Please refer to the online version for the color legend. (**D**) Distribution of community size between communities with and without *MIRs*. (**E**) Degree distribution of *MIRs* and protein-coding genes in communities with size ≥10. See also Supplementary Figures S6 and S8.

We performed enrichment analysis of GO terms on protein-coding genes in the communities. Our results showed that the communities participated functionally in essential biological processes such as cell death, cellular metabolic processes, biosynthetic process, immune system development and cellular response to stress/stimulus (Figure 2A). Moreover, the functional investigation of disease annotations reveals that most of these communities are enriched in *MIRs* involved in cancer and/or hematological disorders (Figure 2B). As observed in the largest community (Figure 2C), disease-related genes (including 10 *MIRs*) tend to form coherent clusters with similar distributions of functional categories. Furthermore, cell-specific or conserved gene interactions are likely organized together. For example, *mir-21* and *TRIM33*, which are both regulators involved in breast cancer (52), are located in an MCF7-specific cluster, whereas *mir-194-2*, *mir-192* and *MEN1* as leukemogenesis (53) in a K562-specific cluster. These observations raise the possibility of functional compartmentalization of chromatin in the nucleus.

We next explored the extent to which *MIRs* are involved in the defined chromatin communities. The largest community, as shown in Figure 2C, involved 896 genes (including 29 *MIRs*). For all the communities, we observed that only a small proportion (27.3%, 201 of 734) of these *MIRs* shared promoters with protein-coding genes. The *MIRs* with shared promoters had similar chromatin features to the *MIRs* with their own isolated promoters (*r* = 0.80 and *P* < 0.001, Mantel test; Supplementary Figure S5B). Remarkably, we found that *MIR*-associated communities are significantly more likely to have a large size (*P* < 2.2 × 10^−^^16^, Wilcoxon's rank sum test; Figure 2D), indicating that *MIRs* demonstrate widespread communication in the transcriptionally active genome, or alternatively, a community with more nodes has a higher chance to overlap with *MIRs*. This notion was further confirmed by the observation that *MIRs* have a larger number of interactions than protein-coding genes (*P* < 3.0 × 10^−^^4^; Figure 2E).

### Systems coordination of chromatin communities through miRNA-target interactions

**Figure 3.**
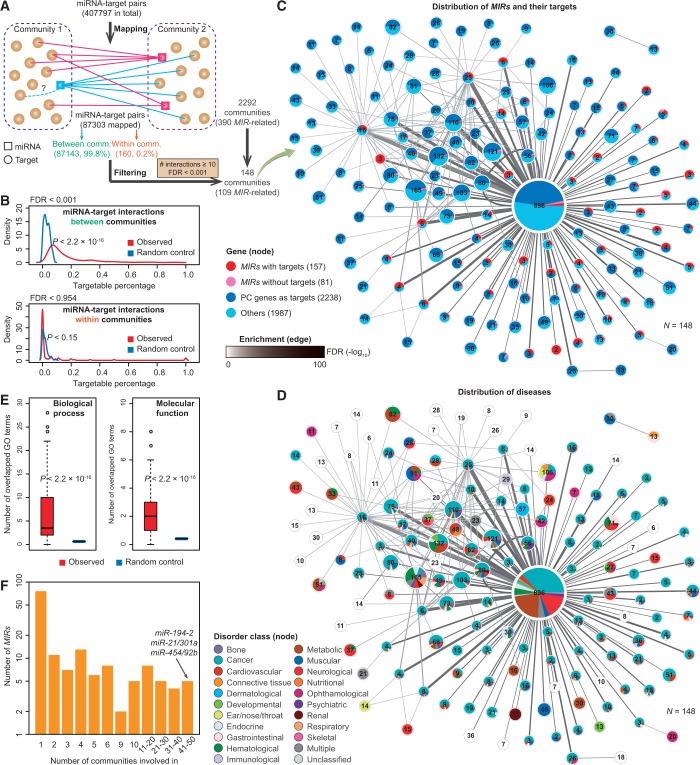
miRNA genes as regulators linking functional-related communities. (**A**) Methodology of association analysis of chromatin communities with miRNA-target interactions. Genome-wide miRNA-target pairs were mapped to chromatin interaction networks. It is notable that, of the mapped pairs, only 0.2% were found within communities. Furthermore, in this investigation, two communities were considered to be linked if there were at least ten miRNA-target interactions between them and the statistical significance of their interactions was tested by a hypergeometric test corrected for multiple comparison by a false discovery rate at 0.001 level. Using the cutoff, a network involving in 148 communities is shown in **C**. (**B**) Distribution of the percentage of genes in a community targeted by miRNA genes (*MIRs*; dark grey). Random control (light grey) with the same number of genes chosen randomly is shown for comparison. FDR (false-discovery rate) value was calculated through permutations (10^3^ randomizations). The Mann-Whitney U test was used to calculated the *P*-value. Analysis was done for miRNA-target interactions between communities (top panel) and within communities (bottom panel). (C and **D**) Networks showing community-community interactions. Each node represents one community. Node size is scaled to the number of genes. Edge line width proportions to the number of miRNA-target pairs between two communities. Edge shading corresponds to the statistically significant of their interactions. (C) Distribution of genes (including *MIRs* and protein-coding genes) within one community is shown in pie chart. (**D**) Distribution of disease class is shown in pie chart. The color denotation of disease classification is taken from (24). Please refer to the online version for details. Nodes with blank indicates no available annotated disease genes. Note that interacting communities shared similar patterns of disease distribution. (**E**) Distribution of the number of shared enrichment GO terms (left panel, biological process; right panel, molecular function) between two linked communities (left bar). As control (right bar), the distribution is calculated with the same number of genes chosen randomly. The *P*-value was calculated by the Mann-Whitney U test. (**F**) Histogram of the number of communities a *MIR* is involved in, identifying the five *MIRs* associated with the largest number of communities. See also Supplementary Figure S9.

Mature miRNAs result in posttranscriptional repression of protein-coding genes (2). So it is reasonable that *MIRs* and their targets should escape to be transcribed together (as to be observed in the same communities). If not, it leads to a paradox, as it is not economical for the cell to first transcribe target genes and then later repress them by *MIRs* produced from the same factory. We thus asked whether miRNA–target pairs result from *MIRs* and their target genes that are produced from the same chromatin communities. We mapped a comprehensive list of high confident miRNA–target pairs to the above described chromatin communities (Supplementary Figure S6B and Supplementary Data Set S5). To our surprise, we found that only 0.2% of the total mapped miRNA–target interactions were found to be from the same communities, while nearly all (99.8%) of interaction pairs were mapped between different communities (Figure 3A). Permutation (10^3^ randomizations) analysis showed that the actual percentage of genes in one community targeted by *MIRs* in another community was significantly higher than that of random control (FDR < 0.001; Figure 3B, Top), while the probability of *MIRs* targeting to genes from the same communities (the percentage of self-targetable) was comparable with that of by chance (Figure 3B, Bottom).

We suspected that *MIRs* may act as the linkers that connect distinct function-related communities via miRNA–target interactions. To test this hypothesis, we first constructed a chromatin-associated community–community interaction network by denoting the chromatin communities as its nodes and statistically significantly (*P*-value from hypergeometric test with correction for multiple comparisons, *P* < 0.001) miRNA–target interactions distributed among these communities as edges (Figure 3A). Here we needed at least 10 miRNA–target interactions mapped to each pair of communities. To be cautious, we performed an extension analysis by relaxing this criterion (for example, at least five interactions), and we found similar results (Supplementary Figure S9A). We, therefore, obtained a network involving 148 communities, 109 of which were *MIR*-related ones (Figure 3C). It is notable that about half of the genes in the communities were targeted by *MIRs* from the linked communities, demonstrating the widespread communication, at the posttranscriptional level, of the transcriptionally active genome. We found that expression of *MIRs* was negatively correlated with expression of their targets, albeit only weakly (Spearman's rank correlation *ρ* = −0.14 for K562 and *ρ* = −0.11 for MCF7), supporting the notion of a fine-tuning role of *MIRs* in gene regulation. Accordingly, genes associated with targets in the communities showed slightly lower expression levels than nontarget genes (Student’s *t*-test *P* < 0.003; average expression levels with RPKM values 19.4 versus 23.4 in K562, and 20.1 versus 21.6 in MCF7; Supplementary Figure S9B).

We next investigated the disease distribution of genes in the interconnected communities. We found that connected communities showed coordinated distribution of genes with disease classes and dominant parts of genes were cancer-related (Figure 3D). Genes associated with the same disorder were proposed to share functional characteristics (24). We now asked whether the genes in connected communities share similar molecular functions and are involved in similar biological processes, as annotated in GO. We calculated the number of enriched GO terms shared among the linked communities (see ‘Materials and Methods’ section), finding significant elevation of homogeneity of GO terms with respect to random controls (Figure 3E). Together, our findings reveal that *MIRs* act as widespread systems regulators that act to coordinate the links between function-related communities, as examples shown in Figure 3F. *MIRs* such as *miR-194-2*, *miR-21*, *miR-301a*, *miR-454* and *miR-92b*, each target nearly one-third of the communities, for example.

### The interactome network of miRNAome in the 3D chromatin space

In the following sections, we focus our analyses on *MIRs* involved in the *MIR*–*MIR* chromatin interaction model, as it provides a structural framework for the study of synergistic transcription regulation of *MIRs*. This set of *MIRs* is nearly one-third (459 of 1523) of currently annotated *MIRs* (Supplementary Figure S8A and B). There were 1260 *MIR*–*MIR* interactions, nearly half (47%) of which are intrachromosomal (Supplementary Figure S10A, Inset). It is notable that these *MIR*–*MIR* interactions are not necessary to arise from the same *MIR* clusters, as we observed only 8.8% (111 of 1260) interactions belong to the clusters (Supplementary Figure S10B).

**Figure 4.**
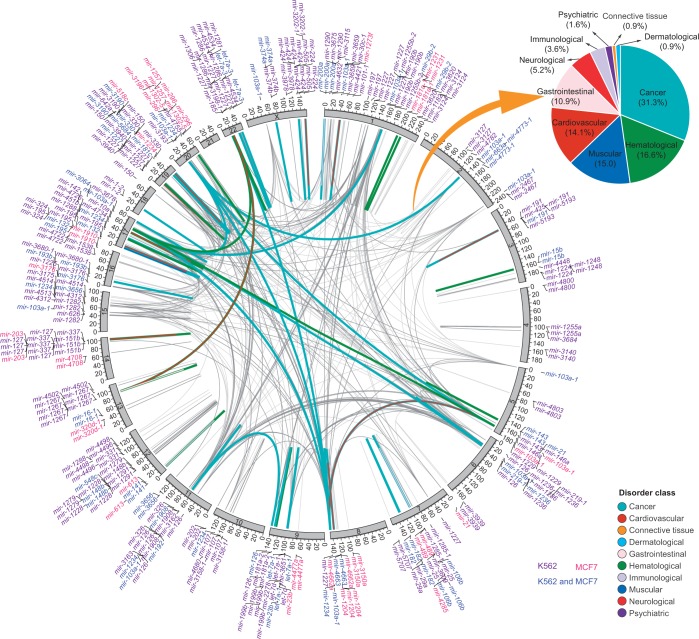
The chromatin contact map of miRNAome. Circos diagram representing disease-related *MIR* chromatin interactome. The linkage of two connected *MIRs* is colored according to their involvement of disease classes (inner track). A pair of *MIRs* may involve in more than more one disorder classes. The color key is shown on the right bottom (see the online version for details). *MIRs* (outer track) are colored according to their cell specificity. Inset pie chart: distribution of *MIR*-related chromatin interactome in ten disorder classes.

The whole *MIR*–*MIR* spatial chromatin interactome is likely to be formed from several hotspot regions, where *MIRs* have strong RNAPII occupancy with high expression levels and are extensively linked through chromatin interactions (Supplementary Figure S10A, Inner tracks). Certain chromosome regions showed enrichment of cell-specific *MIR* interactions. For example, *MIRs* from several chromosome regions, such as 17q22, 17q23 and 20q13 showed MCF7-specific complex interchromosomal interactions, whereas *MIRs* from 1p13, 9q34, 11q12, 13q31, 19p13 and 22q11 showed extensive K562-specific interactions (Supplementary Figure S10A). However, the distribution of *MIR* interactions did not necessarily correlate with their density across the genome, adding evidence to support the view that chromatin interactions of miRNAome are subject to cell-specific regulation. Interestingly, we observed several clusters of disease-related *MIRs* with high expression levels enriched at the hotspot regions (Figure 4). Moreover, nearly half of the interactome are involved in disease classes such as cancer (31.3%) and hematological (16.6%).

**Figure 5.**
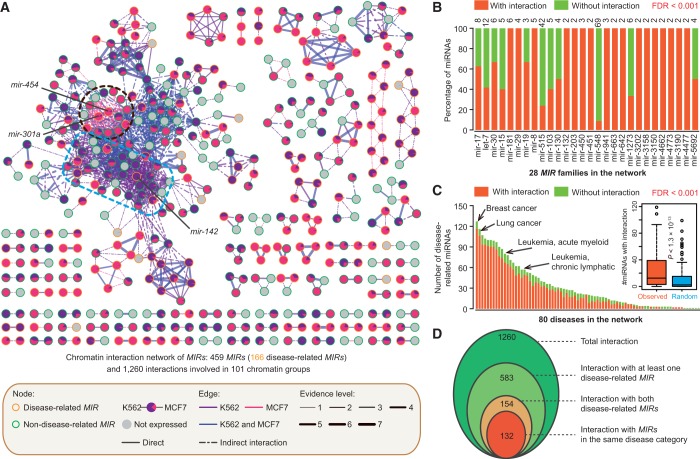
The chromatin interactome networks of miRNAome. (**A**) Network showing chromatin interactions of miRNA genes (*MIRs*). Nodes represent *MIRs* and edges interaction confidence. Nodes are coloured according to their disease states (outline colors) and relative expression values (filled colors), and edge colors indicate whether the chromatin interactions are cell-specific (purple for K562; red for MCF7) or common (blue) between K562 and MCF7 cell lines. Edge size is proportional to the number of independent samples supporting for the interaction. Two largest cell-specific sub-networks are blocked with dashed lines (circle for MCF7 and rectangle K562 cells). The nodes representing three cell-specific *MIRs*, mir-142, mir-301a and mir-454, are indicated. Keys for the network are listed on the box below. Please refer to the online version for details. (**B**) Chromatin interactions between *MIRs* from the same family. The information about *MIR* families was retrieved from miRBase database. The number of *MIRs* from each family is shown on the top of each bar. The percentage of interactions within each family show significant over-representation compared with that of random control. FDR value was calculated through permutations (10^3^ randomizations). (**C**) Distribution of disease-related *MIRs* with chromatin interactions from 80 disease categories. The percentage of interactions within each disease class is over-presented compared to that of by chance (FDR < 0.001). (**D**) Venn diagram depicting the distribution of chromatin interactions based on disease states of the two linked nodes. The number of interactions in each category is listed in each of the components.

The *MIR*-related interactions identified from 3D chromatin space can be viewed from a network perspective (Figure 5A). After mapping expression data and disease information, several insights can be gained from this interactome network. First, the whole network is partitioned into well-demarcated domains (subnetworks) in which *MIRs* showed intensive contact via either common or cell-specific chromatin interactions. Furthermore, *MIRs* with similar expression patterns tended to be colocated, consistent with recent findings that genes involved in chromatin interactions showed correlated expression (22). Moreover, inactive or low-expressed *MIRs* are frequently observed at network boundaries (*MIRs* showing less connection with other *MIRs*) or connections between subnetworks (Figure 5A).

*MIRs* from the same gene family have frequent contact (FDR < 0.001; Figure 5B), supporting the idea that genes with similar functions are spatially organized so as to coordinate transcription. This observation provided direct evidence that *MIRs* from the same family showed co-expression in the derived *MIR* co-expression networks (54–56). We note that the genes for *MIRs* within a specific disease category, such as breast cancer, lung cancer and leukemia, are frequently colocated (FDR < 0.001; Figure 5C). Moreover, *MIR* loci that have spatial interactions tended to be involved in the same disease category. Specifically, of the 154 *MIR*–*MIR* interactions in which both *MIRs* are annotated as disease-related, 132 (86%) have at least one disease category in common (Figure 5D). Taken together, these observations provide evidence that abnormal chromatin conformation and regulation tends to be associated with disease.

### Cell-line specificity of chromatin interactions for *MIR* regulation

To investigate whether the cell-line specificity of chromatin organization correspond to cell-specific expression and the function of *MIRs*, we performed a comparative analysis between K562 and MCF7 cell lines. Of the 1260 *MIR*–*MIR* interaction pairs identified in our analysis, 49 (623) and 10% (124) were specific to K562 and MCF7 cell lines, respectively (Supplementary Figure S8D), suggesting differences in the chromatin architectural context for *MIR* regulation between the two cell types. Accordingly, *MIRs* involved in cell-specific interactions also showed cell-specific expression (Supplementary Figure S8E), implying that cell-specific chromatin organization provides the topological basis for cell-specific *MIR* transcription regulation.

**Figure 6.**
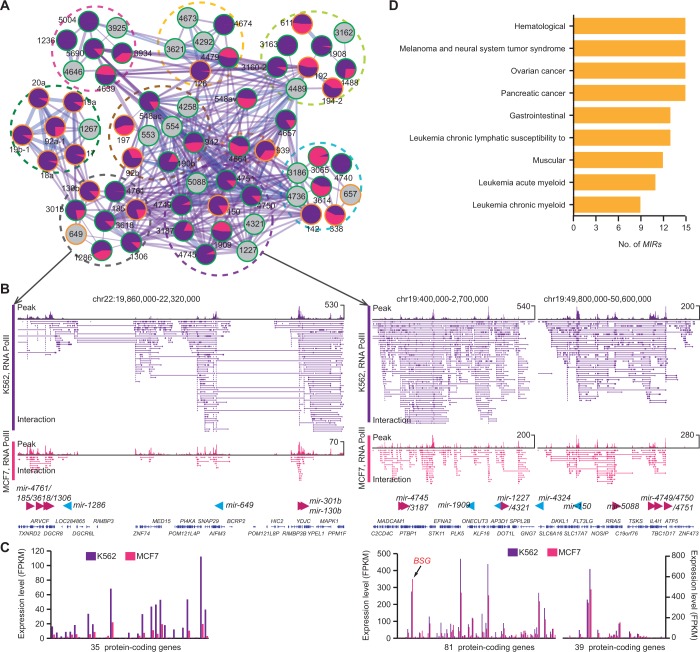
Cell-specific chromatin interactions for miRNA gene regulation. (**A**) Shown are network of K562-specific chromatin-associated miRNA gene (*MIR*) interactions. The expression pattern for each miRNA gene from K562 (dark grey) and MCF7 (middle grey) is depicted as pie chart. The *MIR* identifier is noted for each node. *MIRs* from the same chromosome are clustered with dashed circle. (**B**) Representative examples of chromatin interactions for *MIR* regulation. The tracks for RNAPII binding peaks and interactions from ChIA-PET data are demonstrated. Locations of *MIRs* and surrounding protein-coding genes are shown below the tracks. (**C**) Expression patterns of protein-coding genes surrounding the corresponding regions. (**D**) Significantly enriched disease categories in K562-specific *MIR* interaction network, compared with MCF7-specific network in Supplementary Figure S11.

We focused on the two largest cell-specific subnetworks of *MIR* interactions as indicated in Figure 5A. For the interactions specific to K562 cells, which contained 67 *MIRs* from nine linked chromosomes (Figure 6A), we observed that several leukemia-regulated *MIRs* extensively interacted via spatial chromatin links (Figure 6B), including mir-17-92 cluster (13q31.3, including *mir-17*, *mir-18a*, *mir-19a*, *mir-19b-1*, *mir-20a* and *mir-92a-1*), *mir-126* and *mir-150* (57–63). Remarkably, nearly all the expressed *MIRs* (94%, 46 of 49) were upregulated in K562 cells comparing with MCF7 (Figure 6A). Furthermore, the nearby protein-coding genes showed similar expression patterns with these *MIRs* (Figure 6C). This adds further evidence supporting our view that these two types of genes are organized into the same chromatin architecture and undergo coordinated transcription. We found that the *MIRs* in K562-specific interaction network showed significant enrichment in disease categories such as leukemia, hematological and gastrointestinal-related cancers (Fisher's exact test *P* < 0.1; Figure 6D) when compared with *MIRs* in MCF7-specific network. In the MCF7-specific *MIR* interaction network (Supplementary Figure S11), disease-related *MIRs*, such as *mir-21*, *mir-301a* and *mir-454* have been shown to play a role in the regulation of breast cancer (64–66). Interestingly, these *MIRs* were intensively linked via chromatin interactions with two protein-coding genes, *PPM1D* (67) and *BRIP1* (68,69), which are known to be involved in breast cancer. Altogether, the above results suggest that cell-specifically expressed *MIRs* are subject extensively to cell-specific regulation of the 3D organization of chromatin.

## DISCUSSION

In this study, we have presented an investigation of the relationship between the spatial coordination of chromatin and the transcriptional regulation of *MIRs*. By integrating a large compendium of genome-wide data sets from the ENCODE project (23), including ChIA-PET data, ChIP-seq for various histone modifications, DNA methylation data and RNA-seq data, we have established a link between the 3D spatial organization of chromatin interactions and the expression and function of the human miRNAome. Our work sheds light, at least in part, on a complex landscape of the transcriptional network involving *MIRs*.

Previous studies have demonstrated similarities in the transcriptional regulation of *MIRs* and protein-coding genes. The first evidence comes from the early biological studies demonstrating several instances in which *MIRs* are transcribed by RNAPII (5,17). Additionally, there is evidence that a set of RNAPII-associated transcription factors, such as c-Myc, cAMP-response element binding protein (CREB) and MyoD (70–73), regulate *MIR* expression. Notably, bioinformatics analysis reveal that CpG islands, TATA box sequences, transcription start sites (TSSs), conserved transcription factor binding sites and initiation elements are enriched within the promoter regions of *MIRs* (9,74–78). Consistent with these observations, our data reveal further similarities in the transcriptional properties of *MIRs* and protein-coding genes. We observed with confidence that nearly two-thirds of *MIRs* have RNAPII binding peaks within their annotated promoter regions in the K562 and MCF7 cells (Supplementary Figure S2B). As expected, the binding intensity correlates well with the level of expression (Supplementary Figure S2F–H). Although intergenic and intragenic *MIRs* had distinct patterns of RNAPII occupancy (Supplementary Figure S2D and E), most RNAPII binding peaks are enriched around the proximal TSSs (Supplementary Figure S2C). Furthermore, *MIRs* share consistent patterns of chromatin marks for transcriptional regulation, compared with that of protein-coding genes, which are found to be correlated with RNAPII occupancy in the promoter regions of *MIRs* and are linked to their expression patterns (Figure 1 and Supplementary Figure S5A). We, therefore, conclude that RNAPII serve as the *de facto* polymerase corresponding to *MIR* transcription, implying that both *MIRs* and protein-coding genes share common mechanisms of transcriptional regulation.

We have further constructed a transcription-associated chromatin interaction network, which involves both *MIRs* and protein-coding genes. We observed that discrete gene loci, including *MIRs* and protein-coding genes, from distant regions, were organized into, and co-transcribed from, common spatial domains, referred to as chromatin communities (Figure 2C). We found that such spatially interacting genes showed correlated expression patterns (Figures 5A and 6 and Supplementary Figure S11). Moreover, the communities were enriched in essential cellular functions and include a wide range of *MIRs*. Further investigation based on Hi-C data (29) revealed that these chromatin communities were frequently related to the topological domain structure of the genome (Supplementary Figure S7), suggesting the potential link between topology-associated domains and transcriptional regulation in the human genome.

We found that while miRNA–target interactions were significantly enriched among function-related communities, the *MIRs* and target genes tend to avoid coming from the same spatial community (Figure 3B). We suspect that the community-derived genes (including both *MIRs* and protein-coding genes) may participate in different but functionally related pathways after their transcription. The pathways involved will be likely interrelated, with *MIRs* acting as the fine-tune regulators, which maintain the homeostasis of these processes. *MIRs* in one community may act to switch off expression of target genes from another community, as we observed that targets from certain communities were absolutely switched off (Supplementary Figure S9B). However, we also found that target genes showed even higher expression levels than nontargets within some communities. This observation could be explained by the widespread feedback of regulatory loops involving *MIRs* (79–81). Our data highlight the important roles of *MIRs* in the systems-level coordination of function-related chromatin communities.

We find that genes for *MIRs* from the same disease category tend to be spatially linked together (Figure 5A and C). We suggest that, for at least some *MIR*-associated disorders, the 3D conformation of chromatin is reshaped through chromatin modifications, or mechanical perturbations, that result from changes in the cellular environment. This leads to certain *MIR* loci appearing or disappearing from transcription factories, resulting in the dysregulation of these *MIRs*.

In conclusion, RNAPII-associated ChIA-PET analysis enabled us to deduce a network of spatial interactions involving *MIR* loci, covering nearly one-third members of the whole miRNAome. The results presented here provide a novel insight into the 3D regulation of *MIR* transcription. Based on the similarity of the mechanisms of transcriptional regulation between *MIRs* and protein-coding genes, our results support a context-based transcription factory model in which a context created by the 3D folding of chromatin serves as a general means of coordinating transcription of both *MIRs* and protein-coding genes, highlighting the existence of ‘transcription factories’ in the cell-defined 3D chromatin space for co-transcribing these two types of genes (19). As more data are being generated, we expect that studying the links between spatial chromatin interactions and the miRNAome will enhance our understanding of the mechanisms of *MIR* transcriptional regulation. Our analysis shows that spatial interactions affecting the regulation of the human genome are an important mechanism involved in regulating *MIR* expression in developmental and cellular processes, both in health and disease. This may lead to the discovery of new disease-related *MIR* regulators, with the potential of clinical applications.

## SUPPLEMENTARY DATA

Supplementary Data are available at NAR Online.

## FUNDING

Funding for open access charge: National Natural Science Foundation of China [30971743; 31050110121; 31371328; 31271406 and 31071659] (in part); Federal Office of Agriculture and Food [530-06.01-BiKo CHN to D.J.C., M.C. and C.K.]; Robert Bosch Stiftung [32.5.8003.0116.0 to D.J.C., M.C. and C.K.]; Henry Lester Trust [D.J.C., Z.Z., M.C., A.P.H. and H.P.S.]; Fundamental Research Funds for the Central Universities [M.C. and L.Y.F.].

*Conflict of interest statement*. None declared.

## Supplementary Material

Supplementary Data
