# Supplementary material for: Dissecting the chromatin interactome of microRNA genes
Source: Nucleic Acids Res. 2013 Dec 18;42(5):3028–43. doi: 10.1093/nar/gkt1294 (PMC3950692; doi:10.1093/nar/gkt1294)
Supplement: Supplementary Data [file supp_42_5_3028__index.html]

Dissecting the chromatin interactome of microRNA genes — Dissecting the chromatin interactome of microRNA genes — Supplementary Data 

# Dissecting the chromatin interactome of microRNA genes

## Supplementary Data

files

**Files in this Data Supplement:**

- Supplementary Data - pdf file
- Supplementary Data - xlsx file
