# Supplementary material for: Dissecting the chromatin interactome of microRNA genes
Source: Nucleic Acids Res. 2013 Dec 18;42(5):3028–43. doi: 10.1093/nar/gkt1294 (PMC3950692; doi:10.1093/nar/gkt1294)
Supplement: Supplementary Data [file supp_gkt1294_nar-02745-x-2013-File008.pdf]

SUPPLEMENTARY INFORMATION

Dissecting the chromatin interactome of microRNA genes

Dijun Chen[1,2,3], Liang-Yu Fu[2], Zhao Zhang[1], Guoliang Li[4], Hang Zhang[1], Li Jiang[1], Andrew P. Harrison[5], Hugh P. Shanahan[6], Christian Klukas[3], Hong-Yu Zhang[2], Yijun Ruan[2,4,*], Ling-ling Chen[2,*] and Ming Chen[1,*]

[1]Department of Bioinformatics, College of Life Sciences, Zhejiang University, Hangzhou, 310058, P. R. China

[2]Center for Bioinformatics, Huazhong Agricultural University, Wuhan, 430070, P.R. China

[3]Department of Molecular Genetics, Leibniz Institute of Plant Genetics and Crop Plant Research Gatersleben (IPK), Corrensstrasse 3, D-06466, Gatersleben, Germany

[4]The Jackson Laboratory for Genomic Medicine, Farmington, Connecticut, USA

[5]Department of Mathematical Sciences and School of Biological Sciences, University of Essex, Colchester, Essex, CO4 3SQ, UK

[6]Department of Computer Science, Royal Holloway, University of London, UK

Supplementary Figures 2
Supplementary Tables 14
Supplementary Datasets 17
Supplementary References 17

Supplementary Figures

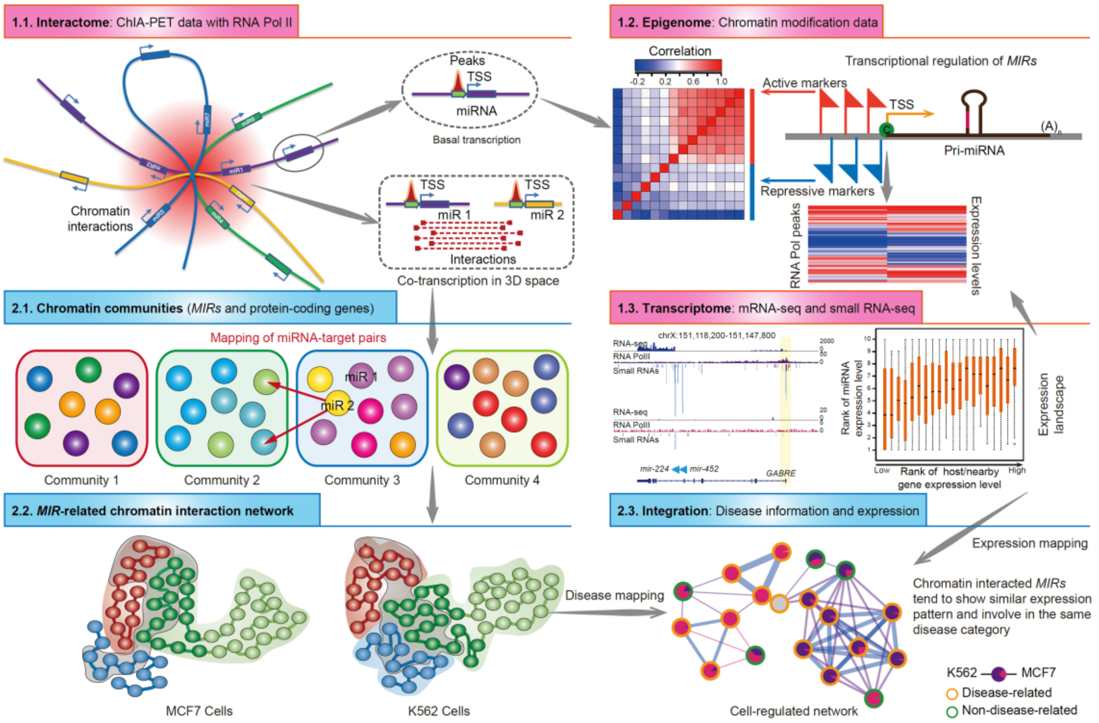

Supplementary Figure S1. Schematic diagram of integrated data analysis strategy in this study.

The sequential analytical steps rely on several types of experimental input (red boxes) and integrated methods (blue boxes). Three main levels of genome-wide datasets were used: (1.1) chromatin interactome data from ChIA-PET with RNAPII, which provided two global datasets: the RNAPII binding sites (oval) and the interactions among the binding sites (rectangle); (1.2) epigenome datasets including histone modifications and DNA methylation data, which were used to depict the chromatin states of miRNA genes (*MIRs*) and their relationship with expression output; (1.3) transcriptome data including mRNA-seq and small RNA-seq data, where were used to measure the expression levels of protein-coding genes and *MIRs*, respectively. Other datasets used in this study can be found in **Supplementary Table S1**. Furthermore, several integrated methods were used to construct chromatin-related *MIR*-*MIR* interaction networks. We first used the ChIA-PET interaction data to construct chromatin interaction networks involving in both *MIRs* and protein-coding genes. miRNA-target pairs were mapped to the defined chromatin communities (2.1; see Materials and methods). We then focused on miRNA-related chromatin interactions and defined a miRNAome chromatin network, which revealed cell-specific chromatin interaction models (2.2). We further integrated RNA-seq data and disease information to character the *MIR* chromatin interaction network (2.3). Data flow involved in this analysis is indicated with gray arrows.

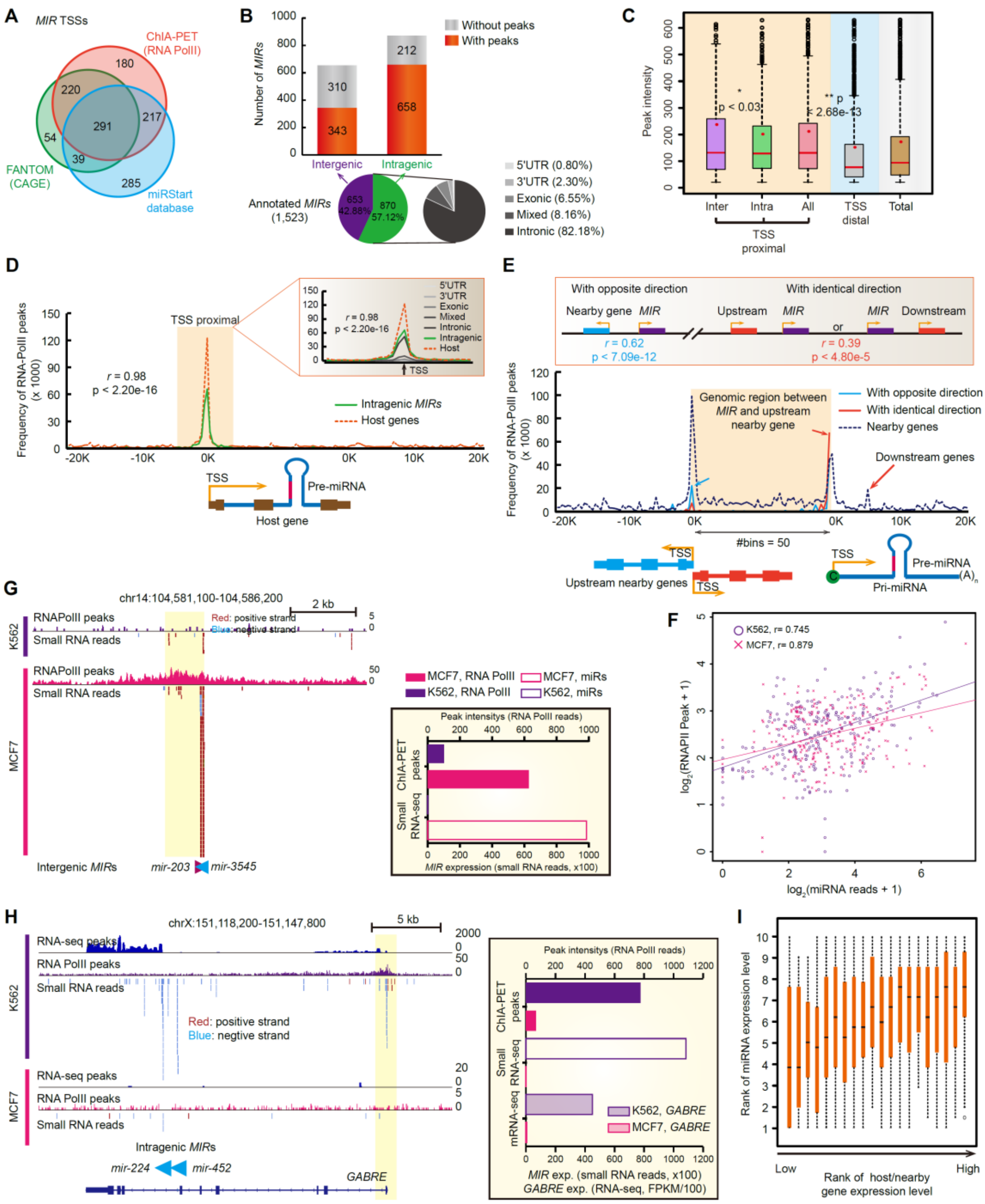

Supplementary Figure S2. Transcriptional regulation of miRNA genes by RNA polymerase II (RNAPII), related to Figure 1.

(***A***) Annotation of transcriptional start sites (TSSs) of miRNA genes (*MIRs*). Venn chart shows the distribution of *MIR* TSSs supported by the three kinds of evidences: miRStart database, FANTOM CAGE tags and RNAPII ChIA-PET peak data. (***B***) Number of *MIRs* with RNAPII peaks around their predicted TSSs (within ±2 kb). Pie chart shows that the most intragenic *MIRs* are from intronic regions of existing protein-coding genes. (***C***) Box plot of RNAPII binding peaks proximal (yellow background, left boxes) or distal (light blue, middle box) to TSS of *MIRs* and the total (gray, right box). Inter, intergenic; Intra, intragenic. (***D***) Distribution of RNAPII peaks around the bodies of intragenic *MIRs* and their host protein-coding genes. Inserted box shows in detail the distribution proximal to *MIR* TSS for each category. (***E***) Distribution of RNAPII binding peaks around the bodies of intergenic

MIRs and their nearby genes. The nearby genes may have either identical (red) or opposite (blue) directions to *MIRs*, as denoted in the upper box. The Pearson's correlations of the distribution of peaks between *MIRs* and the two groups of nearby genes are also shown. (***F***) Correlation of RNAPII binding peaks with transcription levels of *MIRs*. For each *MIR*, the binding intensity was calculated as the total RNAPII reads near TSS (±2 kb) from ChIA-PET data, and the expression level was measured as the total reads from small RNA-seq data. For miRNAs with an identical TSS, their expression levels were summed as a single value. Note that we used only the relatively highly expressed *MIRs* (RPM > 2) for this analysis. (***G***) Representative examples of MCF7-specific regulated *MIRs*, *mir-203* and *mir-3545*. Note that these two *MIRs* are transcribed in cluster from an intergenic region and have their own promoters (yellow background). Inserted plot illustrats the patterns of RNAPII binding peaks and expression levels. (***H***) Representative examples of K562-specific regulated *MIRs*, *mir-224* and *mir-452*. Note that the two *MIRs* are located within the intron of a protein-coding gene, *GABRE*, and these *MIRs* share common promoters (yellow background) with their host gene. Inserted plot demonstratsthe patterns of RNAPII binding peaks and expression levels (measured by small RNA-seq data; two *MIRs* were summed together). As a control, the expression level of *GABRE* (measured by RNA-seq data) is shown as well. (***I***) Box plot illustrating the expression relationship between *MIRs* and their nearby protein-coding genes. The expression values for *MIRs* and protein-coding genes are ranked to ten and twenty levels, respectively. The x-axis shows the twenty groups of protein-coding genes, and the expression levels of corresponding *MIRs* are plotted in box chart for each group. The middle bars represent the mean ranked values.

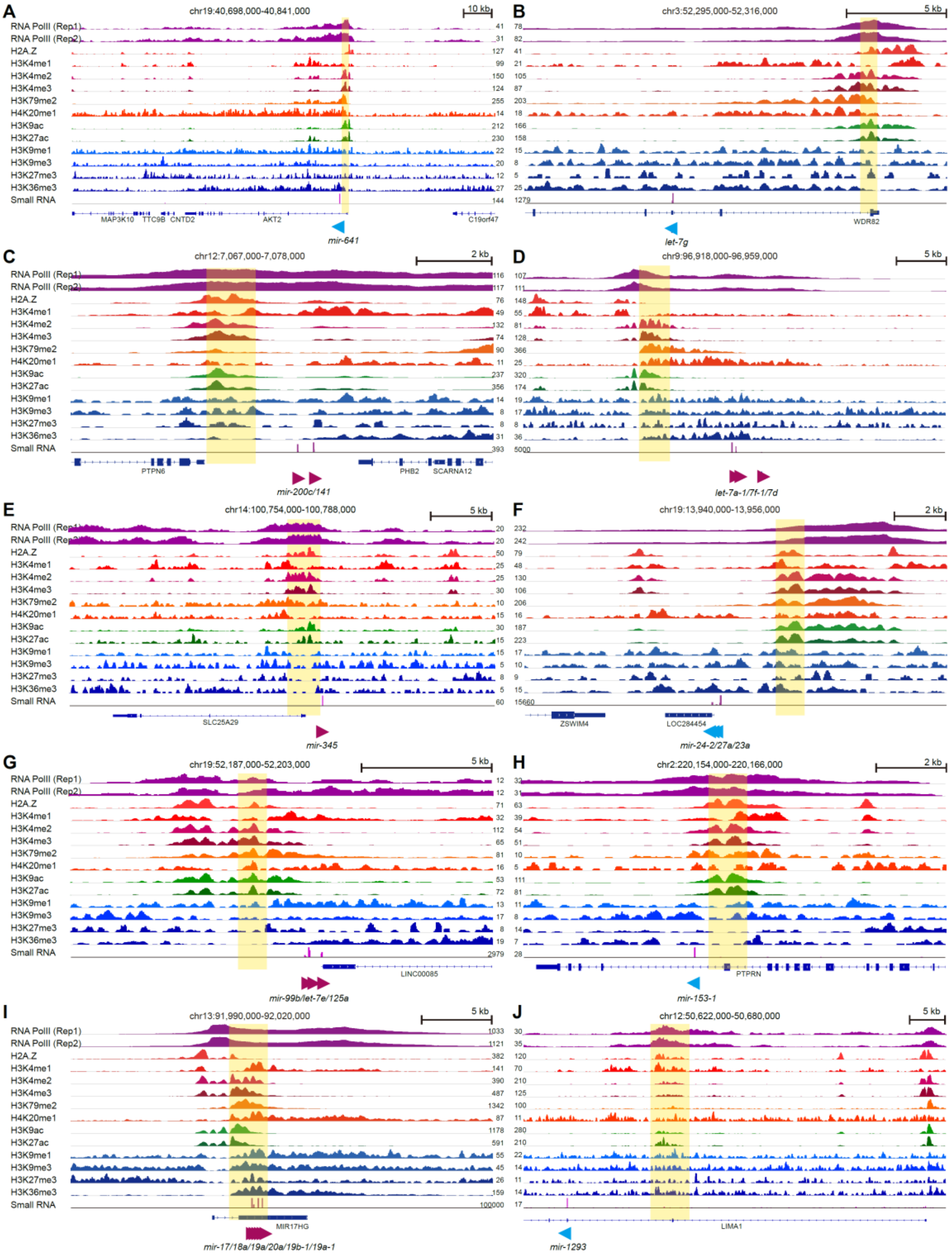

Supplementary Figure S3. Characterization of chromatin signatures around the promoter regions of miRNA genes, related to Supplementary Figure S2.

(***A*** and ***B***) Examples of intragenic miRNA genes (*mir-641* and *let-7g*) share common promoters with their host genes. (***C*** and ***D***) Examples of intergenic miRNA genes (*mir-200c/141* cluster and *let-7a-1/7f-1/7d* cluster) have their own promoters. (***E***, ***F*** and ***G***) Examples of intergenic miRNA genes (*mir-345*, *mir-24-2/27a/23a* cluster and mir-99b/let-7e/125a cluster) share common promoters with their nearby protein-coding genes. (***H***, ***I*** and ***J***) Examples of intragenic miRNA genes (*mir-153-1*, *mir-17* cluster and *mir-1293*) has its own promoter within the

intronic regions of host genes. Note that all data were from K562 cells. Chromosomal locations are indicated on the top. Predicted promoters are highlighted with yellow background.

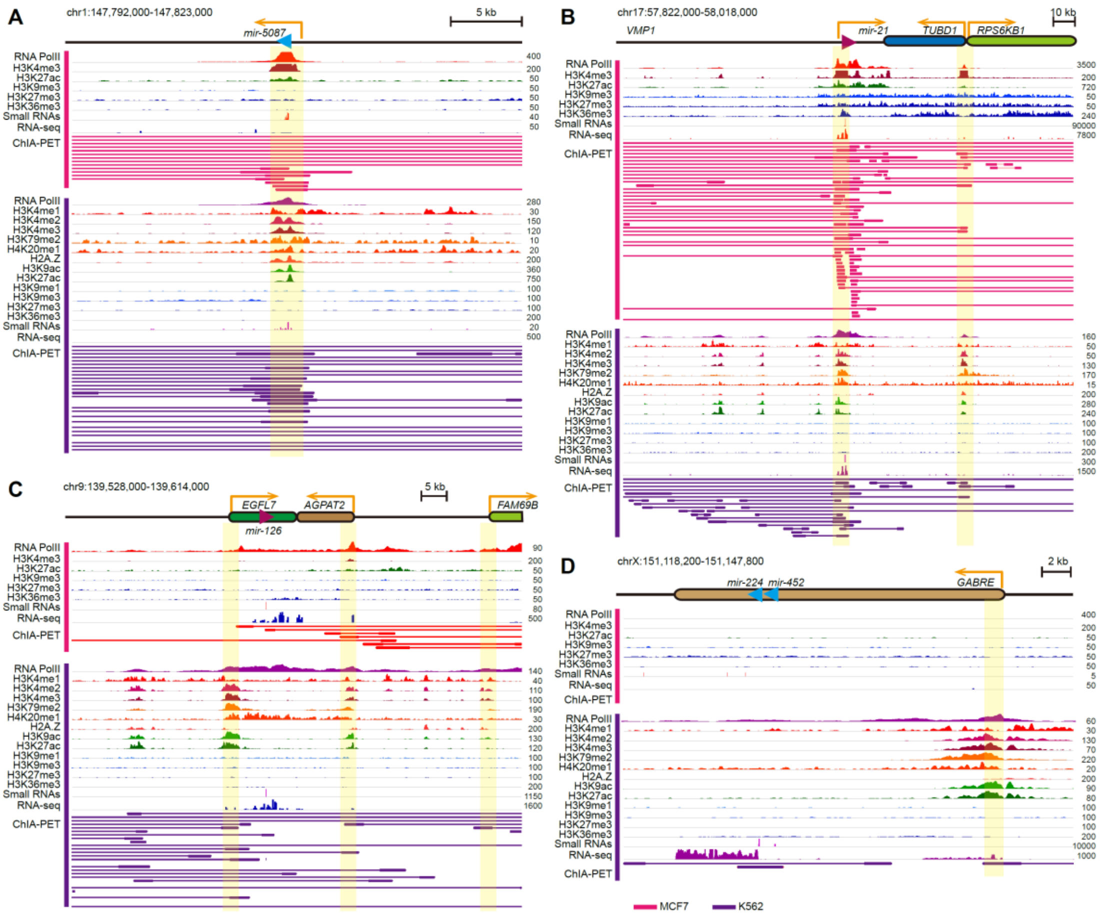

Supplementary Figure S4. Examples of miRNA genes regulated at chromatin level, related to Figure 1. Chromatin features, RNAPII-associated ChIA-PET data and corresponding expression levels are shown for each example. Predicted promoters are highlighted. (***A***) An example of common miRNA genes in both of K672 and MCF7 cells. (***B***) An example of MCF7-specific regulated miRNA genes. (***C*** and ***D***) Examples of K562-specific regulated miRNA genes. Note that miRNA genes shown in ***A*** and ***B*** are intergenic and ***C*** and ***D*** intragenic.

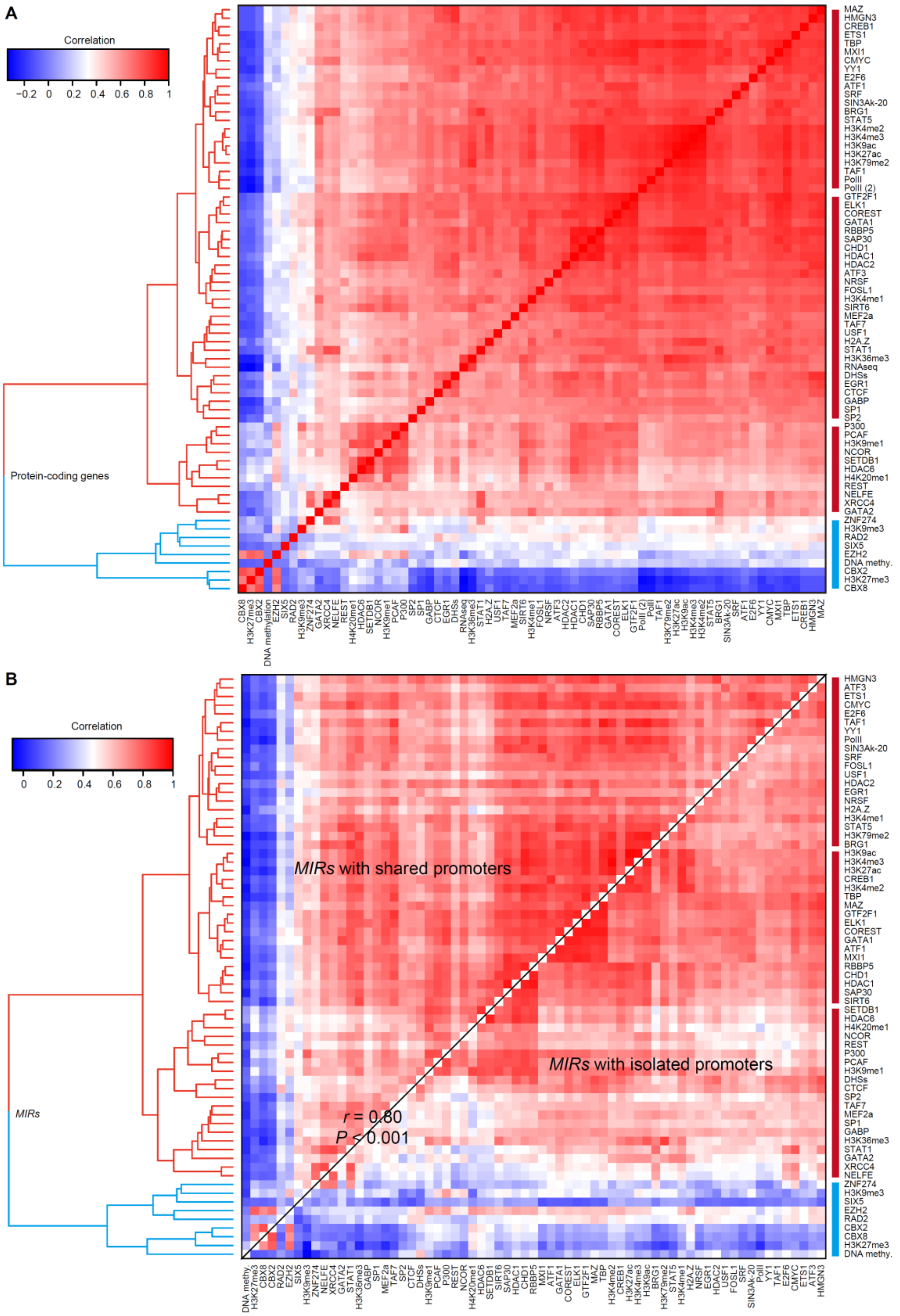

Supplementary Figure S5. Chromatin regulation of miRNA genes, related to Figure 1.

(***A***) Pairwise association analysis of distinct chromatin features for protein-coding genes in K562 cells. Heat map matrix was organized by hierarchical clustering with the tree (left) using Spearman's correlation distances. Different clusters of chromatin features were indicated with color bars (right). (***B***) Pairwise association analysis of

distinct chromatin features for miRNA genes (*MIRs*) in K562 cells. The upper diagonal shows the correlation coefficients based on *MIRs* with isolated promoters. The lower diagonal shows the correlation coefficients based on *MIRs* with shared promoters. Heat map plot was organized by hierarchical clustering with the tree (left) using Spearman's correlation distances based on all *MIRs*, as in **Figure 1*C***. Mantel test (1) was performed on the upper and lower matrices to test their difference (Pearson correlation $r = 0.80$ and $P < 0.001$).

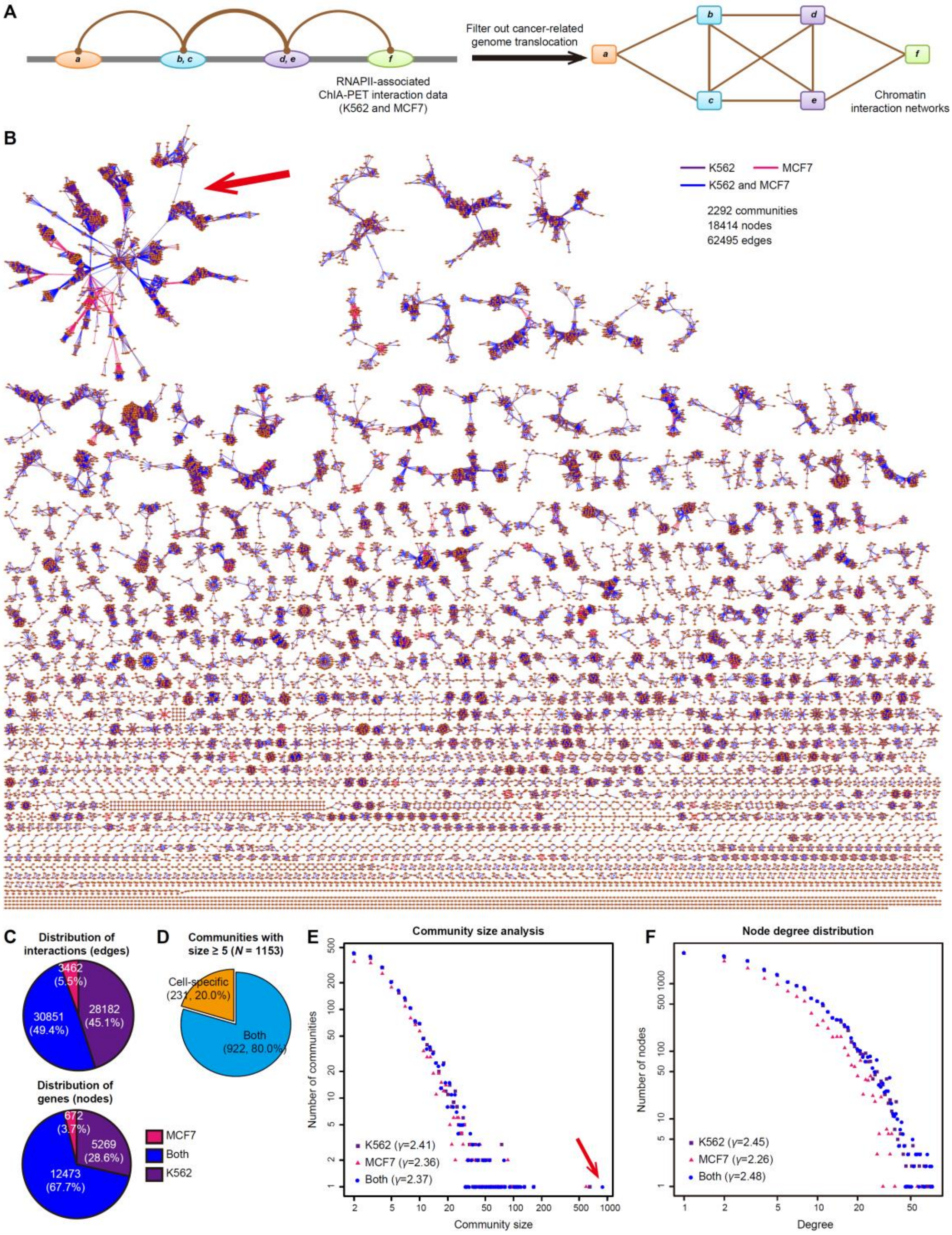

Supplementary Figure S6. Chromatin interaction network and its properties, related to Figure 2.

(***A***) Methodology used to construct chromatin interaction networks. ChIA-PET interactions that overlapped with

existing cancer-related genome translocations were removed before the analysis. Genes (including both miRNA genes and protein-coding genes) within 2 kb from their predicted TSS sites to the two interacting anchor boundaries were considered to be linked each other. Each gene-gene interaction should be validated in at least two separated samples. Analysis was done for K562 and MCF7 cell lines respectively. (***B***) Networks showing the whole chromatin interactions detected from the RNAPII ChIA-PET data. Cell-specific (K562 in purple, MCF7 in red) and common (blue) interactions are indicated with distinct colors. Both miRNA genes and protein-coding genes were included in the analysis. (***C***) Distribution of interactions (edges, left) and genes (nodes, right) in the chromatin interaction network in ***A***. (***D***) Distribution of cell-specific communities and communities shared by K562 and MCF7 cell lines. Only communities with size ≥ 5 were included in this analysis. (***E***) Scale-free size distribution of chromatin community, constructing from K562 (purple), MCF7 (red) and combined (blue) datasets. Red arrow indicates the largest community. (***F***) Scale-free node degree distribution of whole chromatin interaction networks in K562 (purple), MCF7 (red) and combined (blue) datasets.

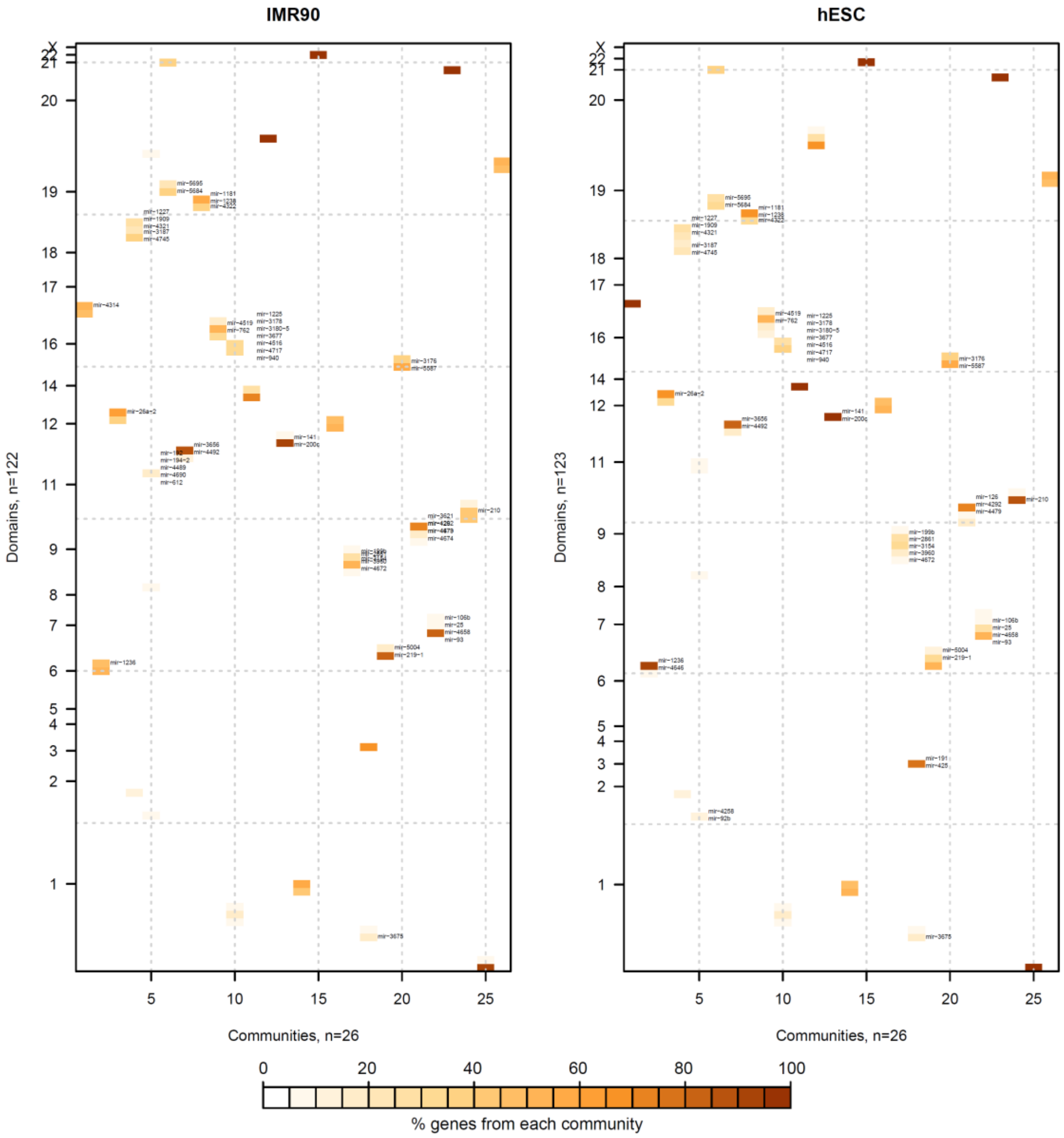

Supplementary Figure S7. Chromatin communities identified by ChIA-PET data are supported by the

topological associated domains (TADs) identified by Hi-C experiments.

Heatmaps show genes from one chromatin community are frequently present TADs identified by Hi-C experiments (2). Rows represent TADs from different chromosomes and columns denote different communities. Percentage of genes from each community in the TADs is shown. miRNA genes are labeled in the corresponding TADs if the percentage value > 10%. For visualization, only communities with size > 50 are shown. Left: TADs from human IMR90 fibroblasts; right: TADs from human embryonic stem (ES) cells. The overall correlation is identical between different cell types.

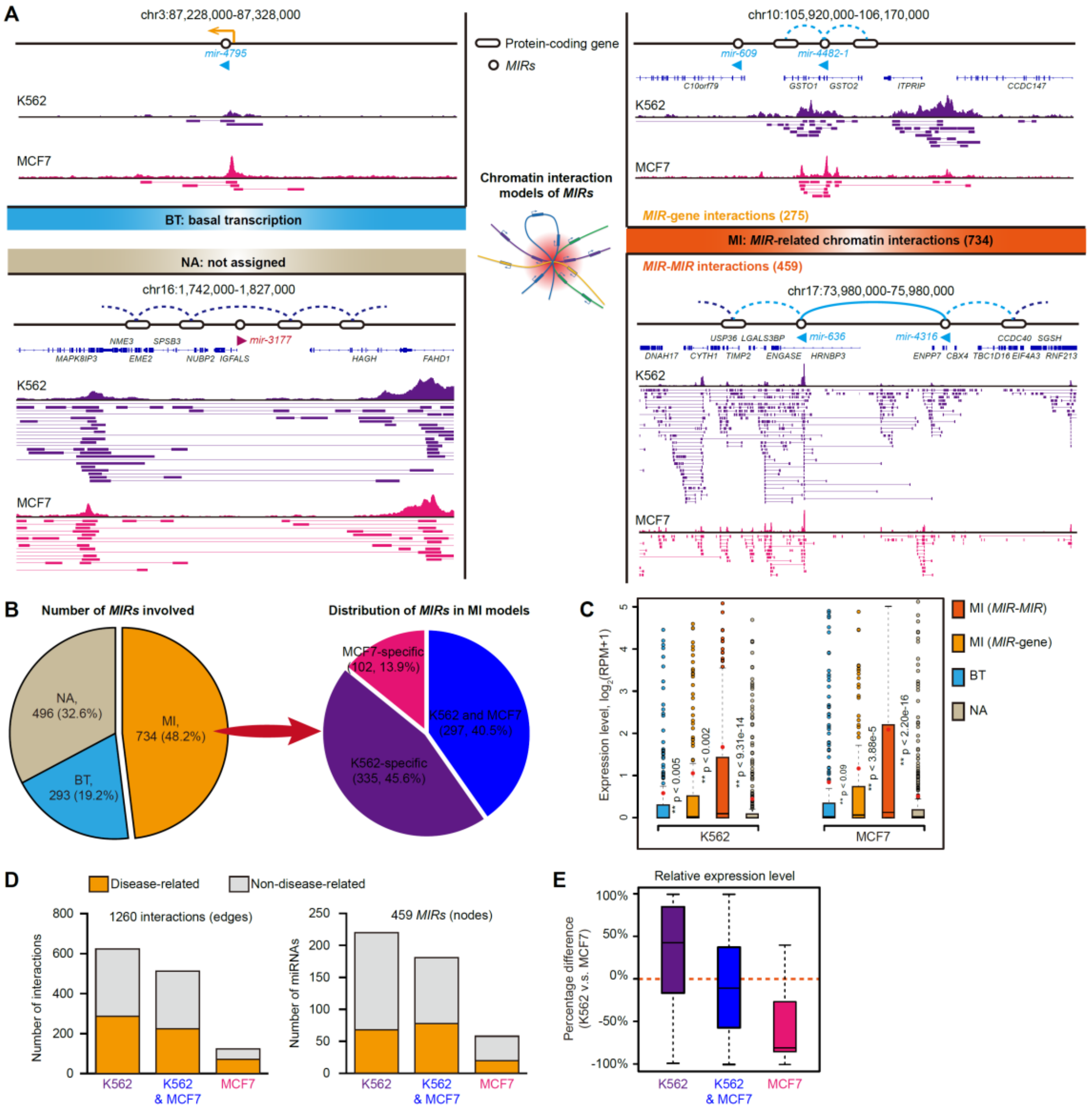

Supplementary Figure S8. Construction of chromatin interaction models of miRNA genes, related to Figure 2.

(***A***) Classification of RNAPII-associated chromatin models for miRNA genes (*MIRs*). All the annotated *MIRs* can be assigned to distinct chromatin interaction models based on how the *MIRs* were involved in the interaction networks: (i) "basal transcription" model (BT), (ii) "*MIR*-related chromatin interaction" model (MI), and (iii) "not assigned" (NA). Representative examples of *MIRs* for each category are shown. The RNAPII peaks and interaction PETs from K562 (purple, track 1) and MCF7 (red, track 2) cell lines are indicated. Solid line, interaction between

MIRs; dashed line, interaction involved protein-coding genes. (***B***) Pie charts showing the number of *MIRs* involved in each chromatin interaction model (left) and the distribution of *MIRs* in MI models. (***C***) Box plot of expression levels of *MIRs* in the chromatin interaction models. Red dot denotes the mean value in each group. Significant p values (t-test) between the mean expressions of genes from different models are indicated. (***D***) Distribution of common and cell-specific chromatin-related *MIR* interactions (left) and *MIRs* (right). Disease-related interaction suggests that at least one *MIR* is annotated as disease-related. Cell-specific *MIR* suggests that all of its involved interactions are cell-specific, otherwise, it is considered as common. (***E***) Box plot demonstrating *MIR* expression patterns among common and cell-specific *MIR* interactions. For each interaction, the percentage difference of relative expression values (K562 versus MCF7) of the involved *MIRs* was calculated.

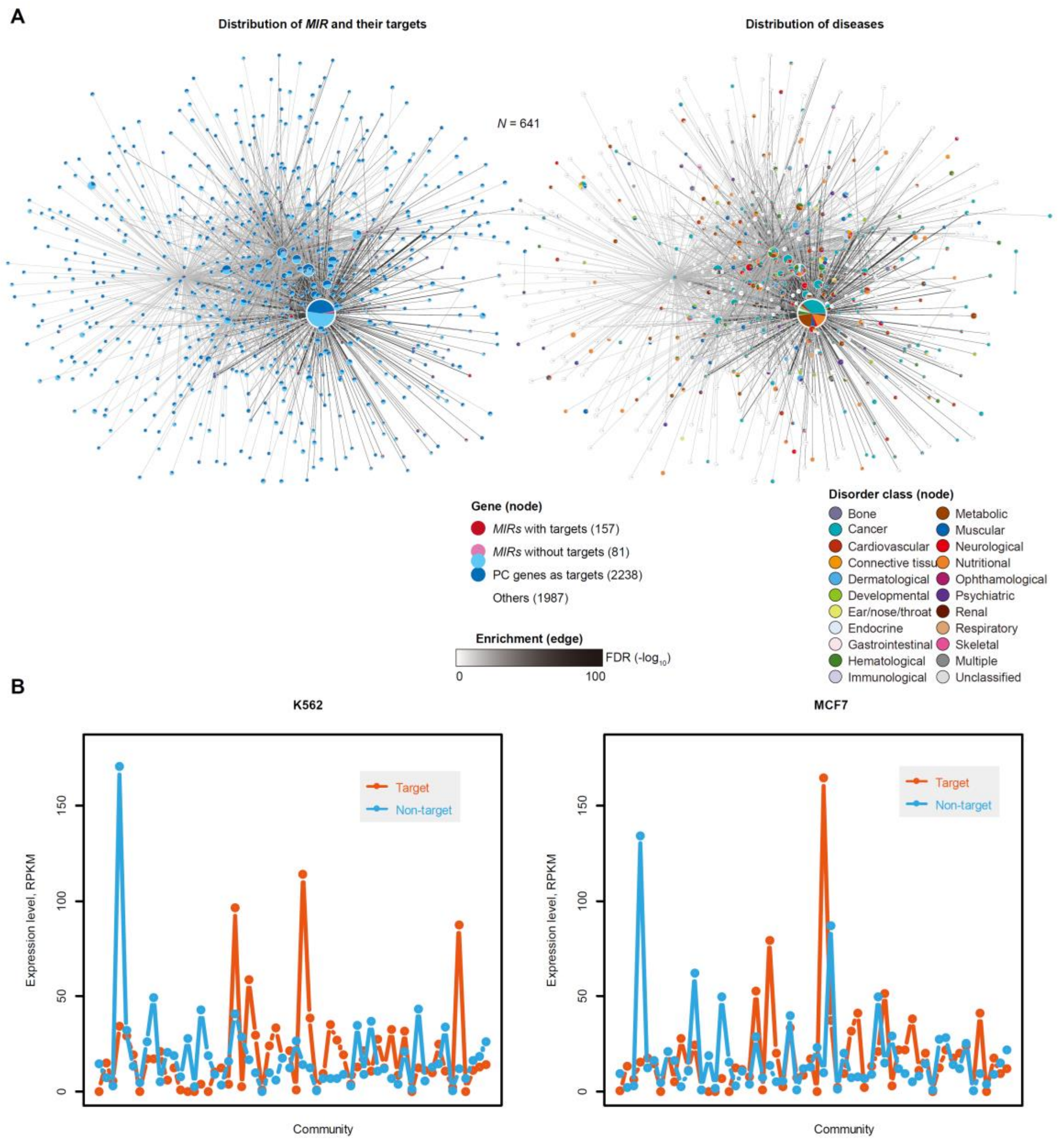

Supplementary Figure S9. Interaction networks of chromatin communities, related to Figure 3.

(***A***) Representation similar to **Figure 3*C*** and ***D***. Each edge should be supported by at least five miRNA-target interactions. Left: distribution of genes (including miRNA genes [*MIRs*] and protein-coding genes) within one

community is shown in pie chart. Right: distribution of disease class is shown in pie chart. Nodes with blank indicates no available annotated disease genes. (***B***) Expression patterns of target or non-target genes in the communities (left for K562 and right for MCF7). Only communities with size ≥ 10 were used in analysis.

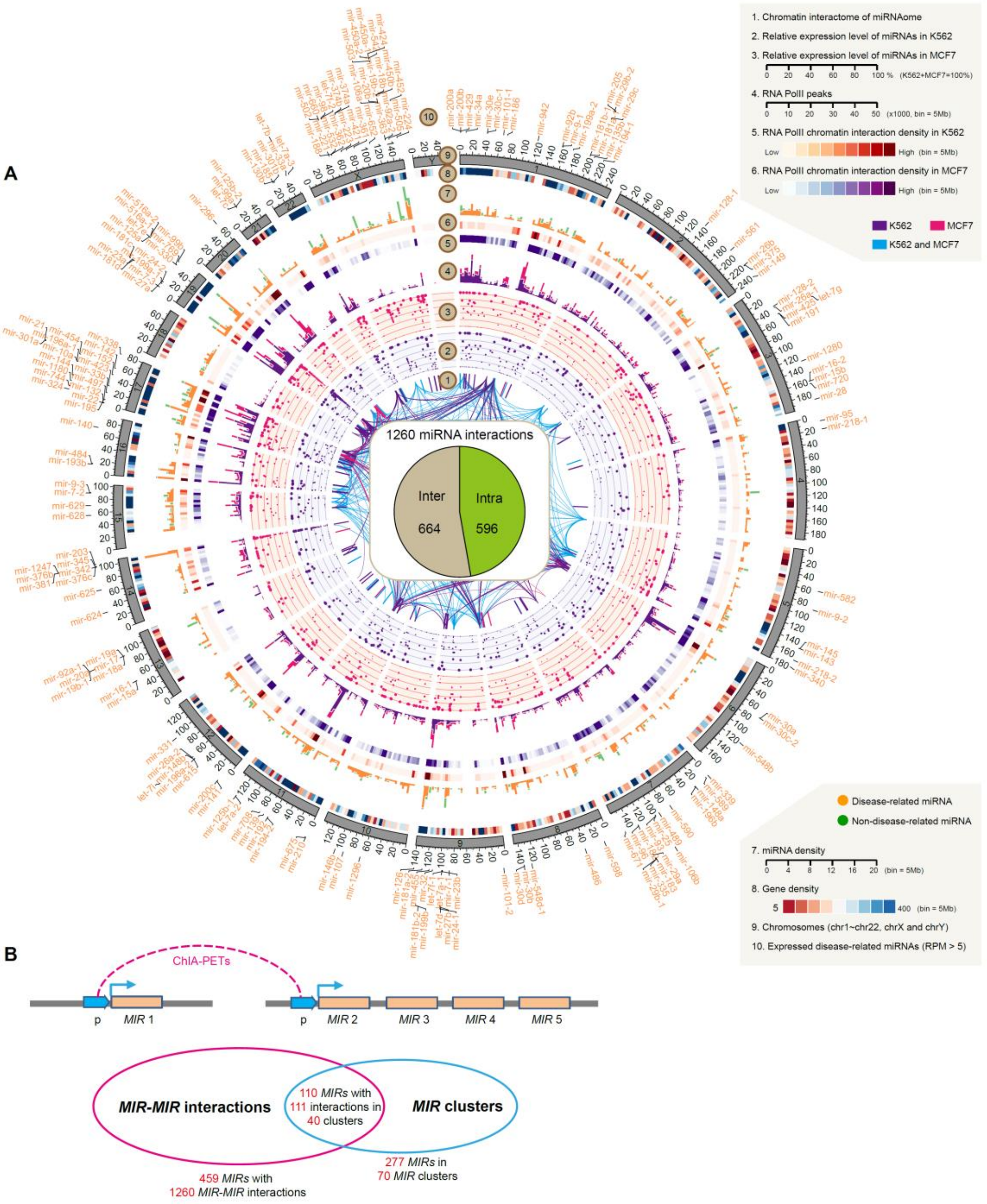

Supplementary Figure S10. The chromatin contact map of miRNAome, related to Figure 4.

(***A***) Circos diagram depicting the miRNA gene (*MIR*)-related chromatin interactome. Two *MIRs* are linked if they showed chromatin interactions supported by ChIA-PET interaction data directly or indirectly (inner links). The whole map involves 1260 interactions. The denotation of each track is labeled on the right side. To show the data, each chromosome was divided into 5 Mb-size windows if necessary, and the data within each window were calculated. Cell-specific (K562, purple; MCF7, red) or common (blue) data related to *MIRs* are colored. Data

tracks are: 1, intra-chromosomal and inter-chromosomal interactions; 2 and 3, relative expression level for each *MIR*, which was normalized to the total expression value from K562 and MCF7; 4, profiles of RNAPII peaks; 5 and 6, density of ChIA-PET interaction data from K562 and MCF7; 7, number of *MIRs* in each window (yellow for disease-related *MIRs* and green for non-disease-related *MIRs*); 8, density of protein-coding genes; 9, the 24 human chromosome (from Chromosome 1 to 22, and X); 10, disease-related *MIRs* (only *MIRs* with RPM > 5 are shown). Inset pie chart: distribution of intra-chromosomal and inter-chromosomal interactions. (***B***) Comparison of *MIR*-*MIR* chromatin interactions and *MIR* clusters. The information about *MIR* clusters was obtained from (3).

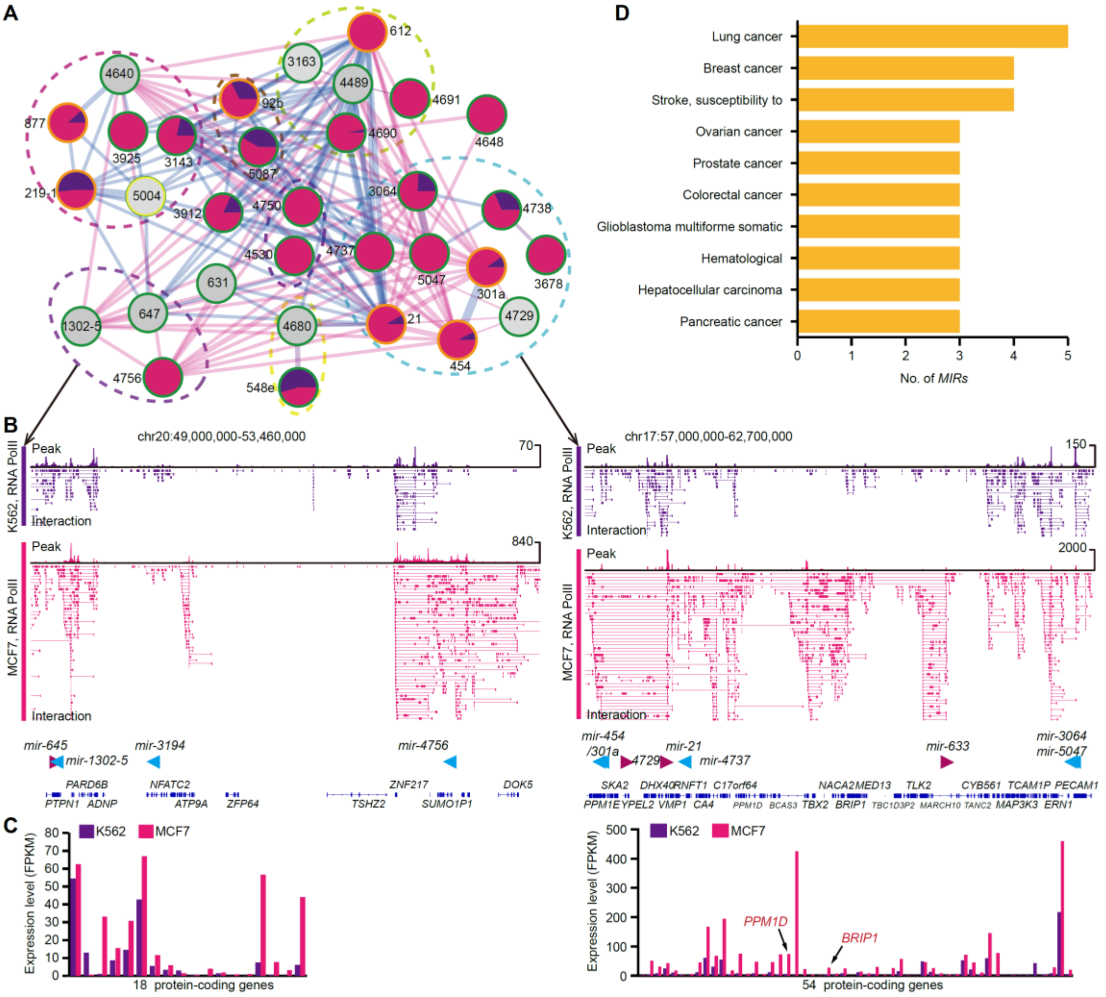

Supplementary Figure S11. Cell-specific chromatin interactions for miRNA gene regulation, related to Figure 6.

(***A***) Shown are network of MCF7-specific chromatin-associated miRNA gene (*MIR*) interactions. The expression pattern for each *MIR* from K562 (purple) and MCF7 (red) is depicted as pie chart. The *MIR* identifier is noted for each node. *MIRs* from the same chromosome are clustered with dashed circle which are coloured according to the color code listed in the cartoon. (***B***) Representative examples of chromatin interactions for *MIR* regulation. The tracks for RNAPII binding peaks and interactions from ChIA-PET data are demonstrated. Locations of *MIRs* and surrounding protein-coding genes are shown blow the tracks. (***C***) Expression patterns of protein-coding genes surrounding the corresponding regions. (***D***) Top ten disease categories that the *MIRs* participated in.

Supplementary Tables

Supplementary Table S1. List of data sources in this study.

**Annotation data:**			
**Category**	**Data source**	**Release/Update**	**URL**
Protein-coding genes	Ensembl and RefSeq	Homo sapiens assembly GRCh37/hg19 (Feb. 2009)<br>Ensembl release 65 (Feb. 2012); RefSeq relase March 17, 2011	http://www.ensembl.org/; ftp://ftp.ensembl.org/pub/release-65/;<br>http://www.ncbi.nlm.nih.gov/RefSeq/; ftp://ftp.ncbi.nih.gov/refseq/H_sapiens/RefSeqGene/
zmicroRNAs (miRNAs)	miRBase	Release 18; November 2011	http://www.mirbase.org/; ftp://mirbase.org/pub/mirbase/18/
Disorder	OMIM	December 2012	http://omim.org/
miRNA disease	PhenomiR /miR2Disease	-	http://mips.helmholtz-muenchen.de/phenomir/; http://www.mir2disease.org/
**TSS-relevant data:**			
**Database**	**Release**	**URL**	**Note**
miRStart	-	http://mirstart.mbc.nctu.edu.tw/	This study identified 847 human miRNA TSSs based on miRBase release 15.
FANTOM web resource	FANTOM4	http://fantom.gsc.riken.jp/	A rich resource that provides DeepCAGE tags to study transcriptional landscape in mammalian genomes.
GEO/ENCODE	-	http://www.ncbi.nlm.nih.gov/geo/query/acc.cgi?acc=GSE33664; Supplemental data from Li et al., 2012; Cell 2012 Jan 20;148(1-2):84-98.	RNA Pol II binding peaks that can be used for TSS identification.
**ChIA-PET data:**			
**Cell type**	**Database**	**URL**	**Datasets**
MCF7	GEO/ENCODE	http://www.ncbi.nlm.nih.gov/geo/query/acc.cgi?acc=GSE33664<br>http://hgdownload.cse.ucsc.edu/goldenPath/hg19/encodeDCC/wgEncodeGisChiaPet/	GSM832456, GSM832457, GSM832458, GSM832459
K562			GSM832463, GSM832464, GSM832465

**ChIP-seq data:**			
**Cell type**	**Database**	**URL**	**Datasets**
MCF7	ENCODE	http://hgdownload.cse.ucsc.edu/goldenPath/hg19/encodeDCC/wgEncodeBroadHistone/ http://hgdownload.cse.ucsc.edu/goldenPath/hg19/encodeDCC/wgEncodeSydhHistone/	CTCF, DHSs, EGR1, FOSL2, FOXM1, GABP, GATA3, H3K27ac, H3K27me3, H3K36me3, H3K4me3, H3K9me3, HDAC2, NRSF, P300, RNA Pol II, RAD2, RNAseq, SIN3a, SRF, TAF1, TCF1, ZNF217
K562			ATF1, ATF3, BRG1, CBX2, CBX8, CHD1, CMYC, COREST, CREB1, CTCF, DHSs, E2F6, EGR1, ELK1, ETS1, EZH2, FOSL1, GABP, GATA1, GATA2, GTF2F1, H2A.Z, H3K27ac, H3K27me3, H3K36me3, H3K4me1, H3K4me2, H3K4me3, H3K79me2, H3K9ac, H3K9me1, H3K9me3, H4K20me1, HDAC1, HDAC2, HDAC6, HMGN3, MAZ, MEF2a, MXI1, NCOR, NELFE, NRSF, P300, PCAF, RNA Pol II, RAD2, RBBP5, REST, RNAseq, SAP30, SETDB1, SIN3a, SIRT6, SIX5, SP1, SP2, SRF, STAT1, STAT5, TAF1, TAF7, TBP, USF1, XRCC4, YY1, ZNF274
**RNA-seq data:**			
**Cell type**	**Database**	**URL**	**Datasets**
MCF7	ENCODE	http://hgdownload.cse.ucsc.edu/goldenPath/hg19/encodeDCC/wgEncodeCaltechRnaSeq/	wgEncodeCaltechRnaSeqK562R2x75Il200GeneGencV3cRep1V3.gtf.gz, wgEncodeCaltechRnaSeqK562R2x75Il200GeneGencV3cRep2V3.gtf.gz
K562			wgEncodeCaltechRnaSeqMcf7R2x75Il200GeneGencV3cRep1V3.gtf.gz, wgEncodeCaltechRnaSeqMcf7R2x75Il200GeneGencV3cRep2V3.gtf.gz, wgEncodeCaltechRnaSeqMcf7R2x75Il200GeneGencV3cRep3V3.gtf.gz
**Small RNA-seq data:**			
**Cell type**	**Database**	**URL**	**Datasets**
MCF7	ENCODE	http://hgdownload.cse.ucsc.edu/goldenPath/hg19/encodeDCC/wgEncodeCshlShortRnaSeq/	wgEncodeCshlShortRnaSeqMcf7CellShorttotalTapAlnRep1.bam, wgEncodeCshlShortRnaSeqMcf7CellShorttotalTapAlnRep2.bam
K562			wgEncodeCshlShortRnaSeqK562CellShorttotalTapAlnRep1.bam, wgEncodeCshlShortRnaSeqK562CellShorttotalTapAlnRep2.bam
**DNA methylation data:**			

Cell type	Database	URL	Datasets
MCF7	ENCODE	http://hgdownload.cse.ucsc.edu/goldenPath/hg19/encodeDCC/wgEncodeHaibMethylRrbs/	wgEncodeHaibMethylRrbsMcf7DukeSitesRep1.bed.gz, wgEncodeHaibMethylRrbsMcf7DukeSitesRep2.bed.gz
K562			wgEncodeHaibMethylRrbsK562HaibSitesRep1.bed.gz, wgEncodeHaibMethylRrbsK562HaibSitesRep2.bed.gz
**miRNA target databases:**			
**Database**	**Release**	**URL**	**Note**
TargetScan	Version 6.2; June 2012	http://www.targetscan.org/	TargetScan predicts biological targets of miRNAs by searching for the presence of conserved 8mer and 7mer sites that match the seed region of each miRNA.
miRanda	40391	http://www.microrna.org/microrna/getDownloads.do	miRanda is an algorithm for the detection of potential microRNA target sites in genomic sequences.
miRDB	Release 4.0; January 2012	http://mirdb.org/miRDB/download.html	miRDB is an online database for miRNA target prediction and functional annotations.
PicTar	-	http://pictar.mdc-berlin.de/	PicTar is an algorithm for the identification of microRNA targets.
DIANA microT	v4.0	http://diana.cslab.ece.ntua.gr/DianaTools/index.php?r=microtv4/index	DIANA microT 4.0 is a program for miRNA target prediction which is based on Artificial Neural Networks. It may be used to search for target genes of annotated or user defined miRNA sequences.
MicroCosm	Version 5	http://www.ebi.ac.uk/enright-srv/microcosm/htdocs/targets/v5/	MicroCosm Targets (formerly miRBase Targets) is a web resource developed by the Enright Lab at the EMBL-EBI containing computationally predicted targets for microRNAs across many species.
**Disease annotation:**			
**Database**	**Release**	**URL**	**Note**
miR2Disease[1]	March 2011	http://www.mir2disease.org/	A manually curated database, aims at providing a comprehensive resource of miRNA deregulation in various human diseases.
PhenomiR[1]	v2.0	http://mips.helmholtz-muenchen.de/phenomir/	The PhenomiR database provides information about differentially regulated miRNA expression in diseases and other biological processes.
OMIM	December 2012	http://omim.org/	Database of the Online Mendelian Inheritance in Man. Only entries with the "(3)" tag were considered.

Note: [1]The combined dataset can be found in **Supplementary Dataset S6**.

Supplementary Datasets

Supplementary Dataset S1. List of miRNA gene TSSs.

Supplementary Dataset S2. List of miRNA genes with RNAPII binding peaks.

Supplementary Dataset S3. Chromatin communities.

Supplementary Dataset S4. Chromatin models of miRNA genes.

Supplementary Dataset S5. miRNA-target interactions in chromatin communities.

Supplementary Dataset S6. miRNA-disease associations.

Supplementary References

1. Mantel, N. (1967) The detection of disease clustering and a generalized regression approach. *Cancer research*, **27**, 209-220.
2. Dixon, J.R., Selvaraj, S., Yue, F., Kim, A., Li, Y., Shen, Y., Hu, M., Liu, J.S. and Ren, B. (2012) Topological domains in mammalian genomes identified by analysis of chromatin interactions. *Nature*, **485**, 376-380.
3. Chien, C.H., Sun, Y.M., Chang, W.C., Chiang-Hsieh, P.Y., Lee, T.Y., Tsai, W.C., Horng, J.T., Tsou, A.P. and Huang, H.D. (2011) Identifying transcriptional start sites of human microRNAs based on high-throughput sequencing data. *Nucleic Acids Res*, **39**, 9345-9356.
